# *RAD50* Is Required for Efficient Initiation of Resection and Recombinational Repair at Random, γ-Induced Double-Strand Break Ends

**DOI:** 10.1371/journal.pgen.1000656

**Published:** 2009-09-18

**Authors:** Jim Westmoreland, Wenjian Ma, Yan Yan, Kelly Van Hulle, Anna Malkova, Michael A. Resnick

**Affiliations:** 1 Chromosome Stability Section, National Institute of Environmental Health Sciences, National Institutes of Health, Research Triangle Park, North Carolina, United States of America; 2 Biology Department, Indiana University–Purdue University, Indianapolis, Indiana, United States of America; The University of North Carolina at Chapel Hill, United States of America

## Abstract

Resection of DNA double-strand break (DSB) ends is generally considered a critical determinant in pathways of DSB repair and genome stability. Unlike for enzymatically induced site-specific DSBs, little is known about processing of random “dirty-ended” DSBs created by DNA damaging agents such as ionizing radiation. Here we present a novel system for monitoring early events in the repair of random DSBs, based on our finding that single-strand tails generated by resection at the ends of large molecules in budding yeast decreases mobility during pulsed field gel electrophoresis (PFGE). We utilized this “PFGE-shift” to follow the fate of both ends of linear molecules generated by a single random DSB in circular chromosomes. Within 10 min after γ-irradiation of G2/M arrested WT cells, there is a near-synchronous PFGE-shift of the linearized circular molecules, corresponding to resection of a few hundred bases. Resection at the radiation-induced DSBs continues so that by the time of significant repair of DSBs at 1 hr there is about 1–2 kb resection per DSB end. The PFGE-shift is comparable in WT and recombination-defective *rad52* and *rad51* strains but somewhat delayed in *exo1* mutants. However, in *rad50* and *mre11* null mutants the initiation and generation of resected ends at radiation-induced DSB ends is greatly reduced in G2/M. Thus, the Rad50/Mre11/Xrs2 complex is responsible for rapid processing of most damaged ends into substrates that subsequently undergo recombinational repair. A similar requirement was found for *RAD50* in asynchronously growing cells. Among the few molecules exhibiting shift in the *rad50* mutant, the residual resection is consistent with resection at only one of the DSB ends. Surprisingly, within 1 hr after irradiation, double-length linear molecules are detected in the WT and *rad50*, but not in *rad52*, strains that are likely due to crossovers that are largely resection- and *RAD50-*independent.

## Author Summary

Double-strand breaks (DSBs) in chromosomal DNA are common sources of genomic change that may be beneficial or deleterious to an organism, from yeast to humans. While they can arise through programmed cellular events, DSBs are frequently associated with defective chromosomal replication, and they are induced by various types of DNA damaging agents such as those employed in cancer therapy, especially ionizing radiation. Elaborate systems have evolved for DSB recognition and subsequent repair, either by homologous recombination or by direct joining of ends. Although much is known about repair mechanisms associated with defined, artificially produced DSBs, there is a relative dearth of information about events surrounding random DSBs. Using a novel, yeast-based system that is applicable to other organisms, we have addressed resection at DSBs, considered a first step in repair. We provide the first direct evidence that cells possess a highly efficient system for recognition and initiation of resection at γ-radiation–induced dirty ends and that the resection is largely dependent on the Rad50/Mre11/Xrs2 complex, identified by the *RAD50* gene. The system provides unique opportunities to address other components in resection and repair as well as to identify the contribution of random DSBs and resection to genome instability resulting from other DNA damaging agents.

## Introduction

Double-strand breaks (DSBs) in chromosomal DNA are common sources of genetic change that may be deleterious or beneficial to an organism. They can arise as direct and processed lesions, errors during replication such as fork collapse, unprotected telomere ends, and they can be integral to programmed developmental processes including meiosis and immunoglobulin rearrangements. The two well-established repair mechanisms, recombination and endjoining, can be distinguished on the basis of requirements for homology (see [1] where the former involves homologous interactions between sister chromatids or homologous chromosomes. A subset of recombinational repair processes involves single strand annealing between homologous regions [2]. Non-homologous endjoining is a highly efficient process in higher organisms and accounts for repair of many kinds of breaks including those that are induced by ionizing radiation [3]. While a great deal is known about the mechanisms of both types of repair using model systems employing single, endonuclease-generated DSBs [1],[4], relatively little is known about the processes associated with repair of random spontaneous DSBs, as might occur during replication fork collapse, or damage-induced DSBs.

Ionizing radiation (IR), a frequently used cancer therapeutic agent, has been traditionally employed as an efficient inducer of DSBs. Based initially on early studies in yeast, there is an elaborate system for recombinational repair of IR-induced breaks whose components extend to humans [5],[6]. For the case of radiation, the ends can be considered as “dirty” in that there is often associated base damage, while in model systems that produce a DSB by an endogenous nuclease (such as HO-endonuclease in yeast), the breaks are “clean” and can even undergo ligation via end-joining processes (reviewed in [7]). Based on a combination of genetics, biochemistry, and repair studies using defined DSBs the early steps in recombinational repair can be broken down into resection of DSBs, binding by single-strand binding proteins, replacement by Rad51 type recombinase proteins, strand invasion and DNA synthesis or an alternative Rad51 independent/Rad52 dependent strand annealing process [8].

For the case of random, multiply induced lesions, as for IR or DSB-inducing chemicals, the presence of breaks is typically signaled by the appearance of H2AX chromatin modification in eukaryotes [9],[10] as well as the development of foci associated with various steps in repair [11]. At the molecular level, the appearance and disappearance of actual DSBs can be detected by changes in populations of large chromosomal DNA molecules using pulsed field gel electrophoresis (PFGE) [12].

Considerable emphasis has been placed on the genetic and molecular events associated with resection. Regardless of the system, the first DNA processing step, originally envisioned for repair of radiation-induced DSBs [13], requires resection of the 5′ end to expose a 3′ strand that could be utilized in strand invasion processes and for priming repair synthesis [14]. Resection at a single defined DSB is strongly dependent on Rad50/Mre11/Xrs2 (MRX) along with the associated activated endonuclease Sae2 [15]. The subsequent extended resection depends on redundant pathways involving Exo 1 or the Sgs1 helicase in combination with the Dna2 long flap-processing nuclease [16],[17] and reviewed in [18].

There is little information about the role of resection at the molecular level in the repair of randomly induced DSB by agents such as IR. In the budding yeast *Saccharomyces cerevisiae*, mutants deficient in the nuclease component of the Rad50/Mre11/Xrs2 complex (MRX) are sensitive to radiation [19],[20]. Moreover, Mre11 nuclease activity may be more important for damaged or blocked ends, such as those linked to Spo11 or hairpin-capped, as compared to clean, HO endonuclease-induced DSBs [8],[21]. While there may be a redundancy in excision by exonuclease I (Exo1), deletion of the *EXO1* gene has little effect on IR sensitivity [19],[20].

To characterize early events in repair of randomly induced DSBs in yeast we sought to develop a system in which the extent of resection (frequency and length of resected ends) at IR induced DSBs could be examined. The challenge was to characterize resection in a population of randomly broken molecules. Previous approaches have relied on the presence of foci associated with resected DNA [11],[22] but lacked resolution. We developed a convenient, robust assay for directly assessing resection following IR induction of DSBs based on our finding that single-strand tails at the ends of large DNA molecules results in a shift to apparent increased MW. The opportunity to follow the fate of radiation-induced DSBs allowed us to characterize the roles of *RAD51*, *RAD52* and *RAD50* as well as *EXO1* in processing ends of randomly induced DSBs and the impact of resection on *RAD50* (*i.e.*, MRX) independent recombination.

## Results

### IR-induced DSBs and chromosome restitution in G2/M-arrested WT, *rad50*, *-51*, and *-52* cells

To investigate molecular events surrounding the processing and repair of DSBs, we examined DSB induction and repair in haploid cells that were arrested by nocadazole in the G2/M phase of the cell cycle. Cells at this stage exhibit resistance to radiation [23] due to opportunities for recombinational repair between sister chromatids [24],[25]. The radiation-induced DSBs can be readily detected in yeast chromosomes by PFGE. There is an approximate random production of DSBs with an efficiency of 0.083 DSBs/mb/krad, which corresponds to ∼2.0 DSBs/per haploid G2 cell per krad. This estimate is based on changes in amounts of unbroken chromosomes of different sizes over the range of 10 to 40 krad (this DSB determination is referred to as “Stained gel, multiple band method” in the Materials and Methods; see example in Figure S1A). A comparable value of 0.053 DSBs/mb/krad over a 10 to 60 krad range was obtained using our recently developed system for measuring break efficiency in a circular Chr III as compared to a linear Chr II, both of which contain a common *LEU2* sequence [26] (see Materials and Methods and Figure S1B). A single DSB in the circular chromosome results in a unique ∼300 kb band (a feature exploited further below). Based on the estimates from the two approaches we assume a value of 0.07 DSBs/mb/krad, which corresponds to ∼140 DSBs/G2 haploid cell at 80 krad.

**Figure 1:**
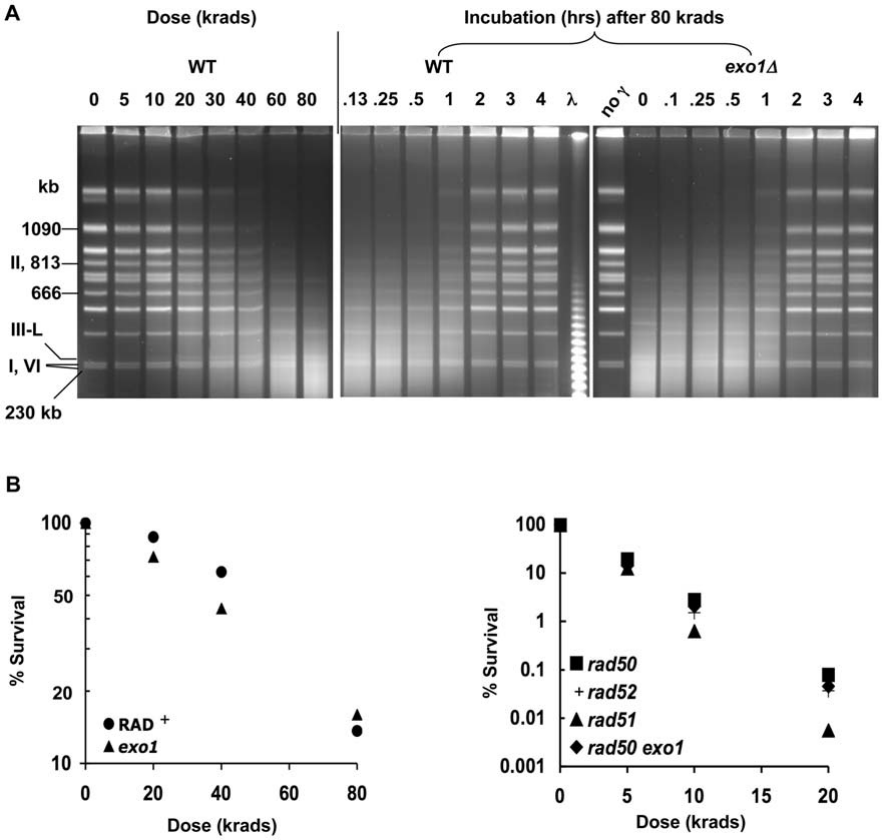
Ionizing radiation–induced DSBs and repair. Haploid cells growing logarithmically in YPDA media were arrested at G2/M with nocodazole and γ-irradiated with 5 to 80 krads from a Cs source. Cells from the 80 krad dose were returned to YPDA containing nocadazole and incubated for up to 4 hours to allow repair of DSBs (A) DSBs and repair in WT and *exo1Δ* strains. DSBs are indicated by the progressive decrease in intensity of linear chromosomal bands and concomitant increase in intensity of a smear of randomly broken DNA with increasing dose (left panel). The linearized circular chromosome III (III-L) containing a single DSB can be seen as a band just above the chromosomes I and VI doublet band starting at 5 krads and increasing in intensity at higher doses. With time, the position of the III-L band appears to shift upward to higher molecular weight (see text). By 2 hrs the band is no longer seen on stained gels due to repair, which results in recircularization and immobilization in the agarose plug. Repair of full-length linear chromosomes is indicated by increasing intensities of chromosomal bands along with decreasing intensity of the smear of broken DNA fragments (center and right panel). The DNAs were run on TAFE gels (see Materials and Methods). (B) Survival of γ-irradiated G2-arrested WT and mutant strains. Cell suspensions in ice-cold water were irradiated and plated to YPDA plates. Results for WT, *rad50Δ*, and *rad52Δ* were from 7 independent experiments; *rad51Δ* from 3 experiments; and *exo1Δ* and *rad50Δ exo1Δ* were from single experiments.

As shown in Figure 1A, postirradiation restitution to full size chromosomes becomes apparent at ∼1 hour after exposure to 80 krads and by 3 hours is ∼90% complete (based on multiple band analysis of pulsed field gels; data not shown) which accounts for the resistance to ionizing radiation (Figure 1B). The induction of DSBs and subsequent repair in G2/M haploid and diploid strains [27] is comparable, suggesting that additional opportunities for recombination provided by homologous chromosomes do not markedly affect the kinetics of repair in G2 arrested cells.

**Figure 2:**
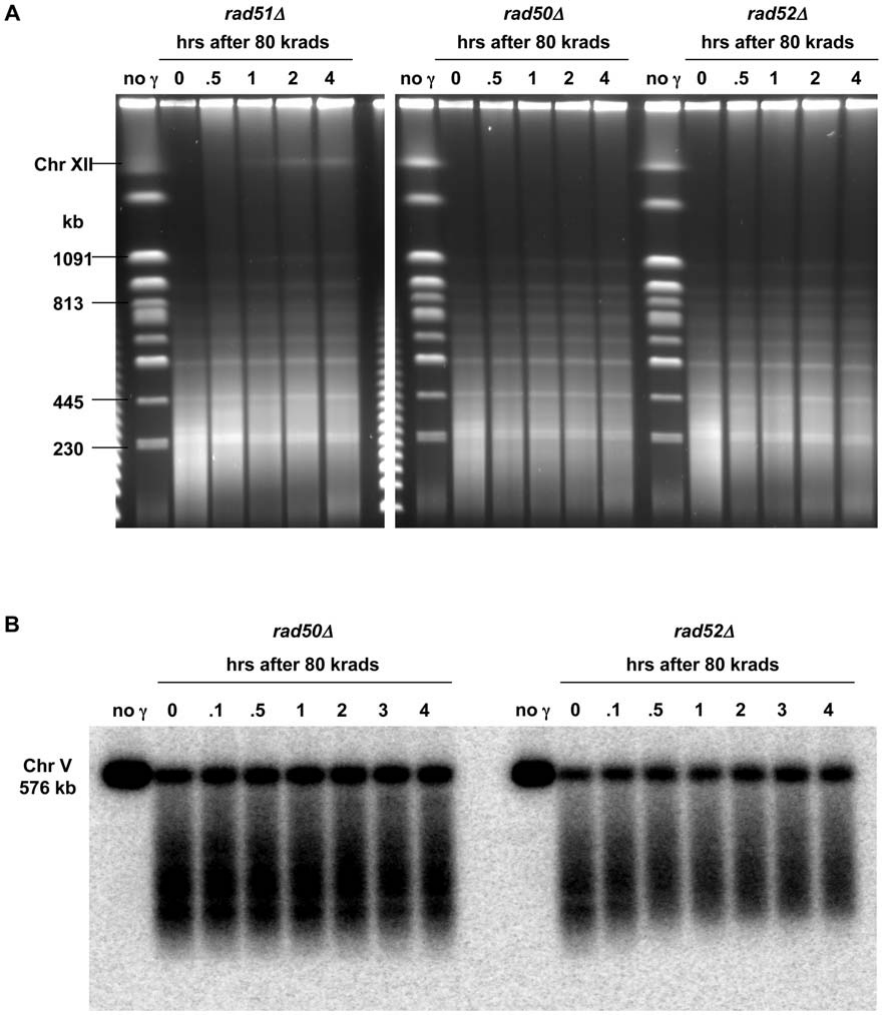
Changes in broken chromosomes following irradiation of HR defective mutants. (A) Absence of repair in HR defective mutants. Logarithmically growing *rad51Δ*, *rad50Δ*, *and rad52Δ* cells were arrested in G2/M with nocodazole, irradiated with 80 krad, and returned to growth medium for up to 4 hrs (as described in Figure 1A). A single break in Chr III resulted in a band above the bottom doublet (230 kb) at 0 hrs. While there was no evidence of repair, the *rad51Δ* strain, unlike *rad50Δ and rad52Δ*, exhibited limited repair of ChrXII, half of which contains ribosomal DNA (first band below the wells). Chromosomal material was displayed by PFGE (CHEF, see Materials and Methods) and the gels were stained with SybrGold. (B) PFGE-shift of broken fragments in *rad52Δ* but not in *rad50Δ*. Cells of *rad50Δ* and *rad51Δ* were arrested in G2/M, irradiated, and returned to growth medium as described in Figure 2A. Samples were run on a pulsed-field gel (TAFE; see Materials and Methods), and a southern transfer was hybridized to a probe (V16) specific for unique sequence near the left telomere of Chr V (16 kb from the end). The lower portion of the smear of broken fragments below the intact full-length Chr V shifts upward by 0.5 hours in the *rad52Δ* but not the *rad50Δ* strain.

There is no detectable restitution of chromosomes in the *rad50Δ*, *rad52Δ*, and *rad51Δ* mutants up to 4 hours after 80 krad exposure of G2/M cells (Figure 2A and analysis of PFGE gels; data not shown). Recombinational mechanisms account for nearly all repair of radiation induced DSBs since a mutation in the *DNL4* gene, required for end-joining ligation, failed to influence the postirradiation status of DSBs or survival in a *rad52* or WT background (data not shown) consistent with results obtained with ku70 rad52 vs rad52 logarithmically growing cells [28]. There appears to be some repair of chromosome XII in the *rad51Δ* mutant, which is presumably due to single-strand annealing repair of resected, ends (see below) involving the ribosomal repeats on this chromosome. The overall lack of restitution of full size chromosomes in the rad^−^ mutants was confirmed by Southern analysis of individual chromosomes following irradiation and incubation (presented in Figure 2B is Chr V). The small amount of restitution in the *rad52* and *rad51* mutants (data not shown) shortly after irradiation is being investigated. While not due to endjoining (data not shown for *dnl4 rad52*), it might be due to repair of closely-opposed single-strand breaks [26].

### Resection identified by PFGE-shift of broken fragments

Surprisingly, for the *rad52Δ* and *rad51Δ* mutants there is a post-treatment decrease in mobility (Figure 2B), referred to as *PFGE-shift*, of the smaller fragment as though there is a general low level of repair (see the lower region of the smear of broken fragments for *rad52Δ* and *rad50Δ* mutants). There was no such shift in the broken chromosomal DNA from the *rad50* mutant up to 4 hours after irradiation and only a small amount at later times.

The apparent increase in size of fragments from the *rad52* mutant (as well as *rad51*), but not *rad50*, during postirradiaton incubation led us to investigate if resection might be involved since the MRX complex can function in initiation of DSB resection as suggested by studies with site-specific DSBs [16],[17]. The resected single strand ends might hamper DNA mobility during its traversal of the gel matrix under pulsed field reorienting conditions, possibly as a result of loss of rigidity or even secondary structures.

**Figure 3:**
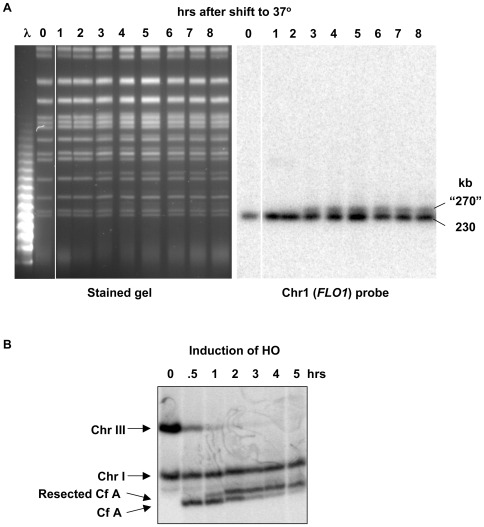
DNA resection leads to reduced mobility (PFGE-shift) of chromosomal size molecules. (A) PFGE-shift of uncapped chromosomes. A temperature-sensitive *cdc13-1* strain which is defective for telomere-capping (DAG760 described in [44]) was grown to stationary phase for 3 days in YPDA media at the 23°C permissive temperature, then diluted 20-fold into fresh YPDA at 23°C and 37°C. Presented are results following the 37°C incubation, a condition that results in 5′ to 3′ resection at telomeres (see text). Samples were collected at the indicated times and prepared for PFGE (TAFE). By 3 hrs, when 97% of cells in the 37°C culture were arrested in G2, novel bands appear in lanes from the 37°C time course at positions corresponding to molecular weights of approximately 25 to 40 kb above many of the known chromosomal bands (left image). The specific chromosomal shifts were confirmed by Southerns using the Chr I specific *FLO1* probe (right image) and other probes specific for chromosomes II, III, V, and VIII (data not shown). These PFGE-shifts were not detected by either stained gels or Southerns for cells incubated at the permissive 23°C temperature (data not shown). (B) PFGE-shift associated with HO-induced DSB. Resection at a unique HO-endonuclease–induced site-specific DSB in Chr III leads to PFGE-shift in broken chromosome fragments. Following growth of WT cells in YEP-lactate media and G2/M arrest in nocodazole for 3 hrs, galactose was added (2% final concentration) to induce HO-endonuclease, resulting in a DSB at *MAT*a (strain AM919). Chromosomes were separated by PFGE. [Because of differences in PFGE-CHEF running conditions for the HO-induced break system, the “apparent” MW shift is different than would be observed for the conditions used in the other PFGE experiments that are described in this paper.] A Southern transfer of the CHEF gel was hybridized with an *ADE1* probe that identifies Chr III and also cross-hybridizes at its native position in Chr I (which also serves as internal standard) [32]. HO cutting was approximately 90% complete by 0.5 hours and PFGE-shift is detected by 1 hour. Note that the unbroken chromosome III contains two copies of *ADE1* inserted at *HML* and *HMR*, which is responsible for the higher intensity of its hybridization to the *ADE1*-specific probe as compared to the Chr I, and Cf A, bearing only one copy of *ADE1*. Also, to achieve the required separation between the resected and unresected fragments, the smaller fragment containing HMR::ADE1 (approximately 100 kb) does not remain in the gel, so that only the larger fragment containing HML::ADE1 is detected.

To test if the PFGE-shift phenomenon could be a result of resection, we examined a mutant deficient in telomere capping, *cdc13-1*. At elevated temperatures, telomeres become uncapped and are recognized by the repair system as DSBs [29]. As shown in Figure 3A, raising the temperature from 23° to 37°C resulted in a marked change in the apparent karyotype with many chromosomes displayed as doublets. Southern analysis of Chr I (Figure 3A) showed that there was, in fact, a doublet consisting of the original chromosome (230 kb) and an apparently larger version (∼270 kb). Similar observations (data not shown) were made with chromosomes I (230 kb), II (813 kb), III (340 kb), V (576) and VIII (565 kb). There appears to be differences in the frequencies of chromosomes shifted, suggesting variation in telomere decapping (Figure 3A).

We also examined the PFGE mobility consequences of resection at a single, unique HO-endonuclease induced DSB on mobility of Chr III fragments. As described in Figure 3B, the original 340 kb chromosome III band disappeared shortly after shifting to galactose and the expected fragment of ∼230 kb was detected. Similar to results with the uncapped telomeres, there was an upward shift in mobility beginning at ∼1 hour. By 2 hours the apparent sizes of most fragments had shifted to ∼240 kb; there was no further change in the apparent size during the subsequent 3 hours. By 2 hours the resection would have been expected to be ∼8 kb (or 4 kb/hr) based on previous studies of resection of HO-induced DSBs [30].

To address PFGE-shift of large molecules directly, we developed an *in vitro* system based on 48.5 kb bacteriophage lambda DNA. As shown in Figure S2A the PFGE migration of the lambda DNA was reduced following treatment with lambda 5′ to 3′ double-strand exonuclease. Resection of 2 kb from each end led to an apparent 50 kb increased size. The system also enabled us to address the impact on mobility of one vs two resected ends (discussed below). One-end and two-end resection fragments were generated by treating lambda DNA with a restriction enzyme (PspOM I) either before or after resection. As shown in Figure S2B, resection from both ends leads to substantially greater PFGE-shift than resection from a single end.

Overall, these findings strongly implicate resected single-strand tails as the source of PFGE-shift of randomly broken chromosomes during post-irradiation incubation.

### Resection of randomly produced single DSBs in circular chromosomes detected by PFGE-shift

**Figure 4:**
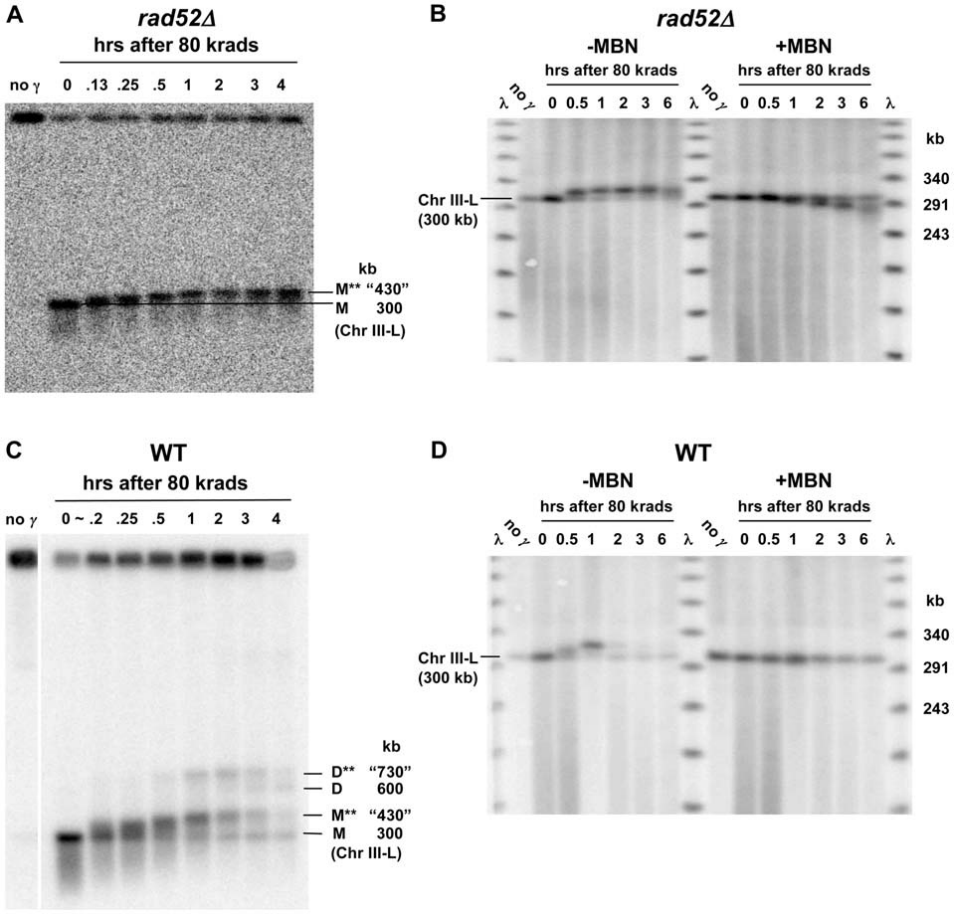
The PFGE-shift in *rad52Δ* and WT resulting from a random γ-induced single DSB is due to resection. (A) Rapid PFGE-shift of broken ChrIII in *rad52Δ* mutant during postirradiation incubation. Nocodazole-arrested G2/M cells were irradiated with 80 krads and returned to YPDA media to allow processing of DSB ends. Presented is a Southern of a pulsed-field gel (TAFE; see Materials and Methods) using a Chr III specific *CHA1* probe. Circular Chr III from unirradiated cells (“no γ”) remained trapped in the plug. Chr III molecules with only one DSB migrate at a position corresponding to 300 kb (“0” hr lane). Less than 10 min after returning cells to YPDA, most of the molecules in the band shifted to an apparent molecular weight that is greater than 300 kb. By 2 hrs the band reached a position of maximum shift corresponding to an apparent MW of approximately “430” kb. Smearing under the linear Chr III band is due to multiple DSBs in Chr III. (B) The PFGE-shift is due to resection based on digestion by mung bean nuclease. PFGE plugs from an experiment involving 80 krad to *rad52*Δ cells and postirradiation incubation (as in Figure 4A) were run on a CHEF gel under conditions to maximize resolution of the linearized 300 kb Chr III (see Materials and Methods). The probe used for this Southern labels the lambda DNA ladder as well as the *LEU2* sequence on Chr III. As seen in the left group of lanes, there is a PFGE-shift of Chr III linearized with a single random DSB within 30 min postirradiation incubation. Duplicates of these plugs were treated with mung bean nuclease to remove 3′ single-strand tails. As shown in the group of lanes on the right, the nuclease treatment resulted in molecules from the ≥0.5 hrs incubation exhibiting faster PFGE mobility. By 6 hrs, the average size of the *trimmed* linearized Chr III was reduced by ∼15–20 kb, corresponding to an average resection rate of ∼1.5 kb per DSB end per hr. (C) Rapid PFGE-shift and appearance of novel bands in WT cells. WT cells were arrested at G2/M by nocodazole, irradiated with 80 krads and returned to YPDA media. Plugs were prepared at the indicated times and run on PFGE (TAFE; see Materials and Methods). The probe used for this Southern corresponded to the *LEU2* sequence on chromosome III, which is circular at the beginning of experiment, but becomes linear following radiation-induced breakage (similar to Figure 4A). In addition to the non-resected (M) and resected (M**) bands seen in *rad52*, two additional bands referred to as D and D** appear within 30 min after irradiation. The D band corresponds to an MW that is twice that of the non-shifted linear monomer. Since the appearance of D and D** are *RAD52*-dependent, these are considered to be recombinant molecules (discussed in text). Band D** is proposed to be a linear dimer with both ends resected. [The “no *γ*” lane is from a different part of the same gel. The DNA in the well of the last lane did not transfer well. The “∼0.2 hr” time point is not precise because EDTA was not added although cells were put on ice. The amount of linear ChrIII detected in the “no *γ*” lane is <3% of that in the well.] (D) Resection in WT cells is the source of the PFGE-shift in ChrIII containing a random γ-induced single DSB. As in Figure 4B, mung bean nuclease treatment of plugs from the 80 krad+repair experiment (right half of image) abolished the PFGE-shift seen with untreated plugs (left half of image). The rapid and efficient repair of resected DSB ends in WT cells prevented accurate measurement of the resection rate.

**Figure 5:**
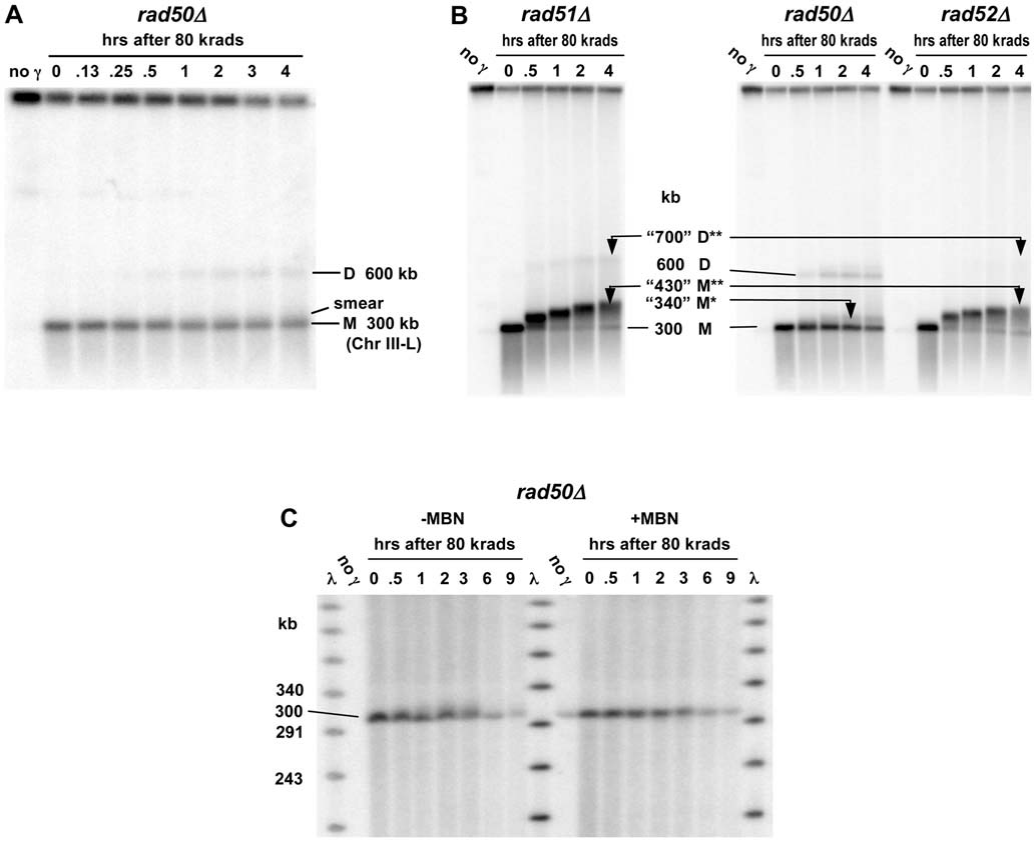
The PFGE-shift associated with a random γ-induced single DSB requires Rad50 (MRX). (A) Limited processing of Chr III DSBs in *rad50Δ*. Nocodazole arrested G2/M cells were irradiated with 80 krads and returned to YPDA media. Plugs were prepared from the indicated time points and run on PFGE (TAFE; see Materials and Methods). Presented is a Southern of the gel using a Chr III specific *CHA1* probe. Circular Chr III from unirradiated cells (“no γ”) remained trapped in the plug. Chr III molecules with only one DSB migrate to a position corresponding to 300 kb (0 hr lane). Bands corresponding to M (linear non-resected monomer) and D (linear non-resected dimer) of Figure 4C (WT 80 krad+incubation) appear with the *rad50Δ* strain. However, bands corresponding to M** or D** of Figure 4C are not seen, indicating that resection is greatly reduced in MRX-deficient cells. The faint smear extending above the linear non-resected M band, detectable by ∼1 hr indicates limited resection and/or resection possibly of only one end of these molecules as discussed in the text. (B) The limited PFGE-shift in *rad50Δ* after 80 krad differs from *rad51Δ* and *rad52Δ*. The smear of broken Chr III (Figure 5A) was characterized by PFGE (CHEF; see Materials and Methods). Comparisons were made with the PFGE shift found with the *rad51Δ* and *rad52Δ* mutants. (The stained gel is presented in Figure 2A.) For *rad50Δ* (center lanes) no band is observed corresponding to the typical M** band seen with *rad51Δ* (left) or *rad52Δ* (right) mutants. Instead, there is a slow accumulation of a faint band (M*) with a PFGE-shift corresponding to ∼40 kb increase in apparent molecular weight. (C) The effect of mung bean nuclease treatment on the mobility of broken Chr III. PFGE plugs from an experiment involving 80 krad to *rad50Δ* and postirradiation incubation (as in Figure 5A) were run on a CHEF gel under conditions that maximize resolution of the linearized 300 kb Chr III (see Materials and Methods). A slight smear is observed above the linearized monomer band in the no MBN lanes (*i.e.*, at 2 and 3 hrs after 80 krads). Mung bean nuclease (+MBN lanes) removes the smear and yields tighter ∼300 kb bands, indicating that the smear is due to resection. However, unlike for *rad52Δ* (Figure 4B), the products of the MBN treatment did not run at a detectably faster rate than the unresected monomer in the 0 hour lane, indicating that little resection had occurred. The “λ” lanes correspond to 48.5 kb lambda DNA ladder.

Based on these results it should be possible to follow the fate of a single, randomly produced DSB in a circular chromosome since this would lead to a unit size linear molecule with two damaged ends (as described in Figure S1B). Within 10 minutes after induction of ∼140 DSBs per genome (80 krad) in G2/M nocadazole-arrested cells, linearized Chr III molecules from the *rad52* and *rad51* cells began to exhibit an apparent increase in size as described in Figure 4A (results with *rad51* are described in Figure 5B), well before repair occurs in WT cells (Figure 1A). In the *rad52* mutant most linearized molecules exhibited a shift by 30 minutes and there was little further shift in PFGE mobility after 1 hr (also see left half of Figure 4B). Nearly all the linearized molecules exhibited a shift by 1 hr, independent of dose (results described below). These results are consistent with the increase in apparent size of the population of broken Chr V molecules in Figure 2B.

To establish that the PFGE-shift of the γ−linearized molecules was actually due to resection, the plugs of *rad52* chromosomal DNA used for PFGE were treated with mung bean nuclease (MBN) to digest single strand tails (see Materials and Methods). As shown in Figure 4B, treatment of chromosomal DNAs from cells that had been incubated after irradiation resulted in reduction in the apparent MW whereas MBN did not change the mobility of γ−linearized molecules at time “0,” consistent with MBN acting only on ssDNA. (The small amount of linear Chr III DNA from the unirradiated cells is somewhat increased by MBN, possibly indicating that some circular molecules in the plugs have single strand regions; the PFGE-CHEF conditions used in these experiments were modified to expand opportunities to see chromosomal changes.) With increased postirradiation incubation, the size of the molecules after MBN was decreased. Based on the average size of molecules after MBN treatment (Figure 4B, right side), resection proceeds at a rate of ∼1.5 to 2 kb/hour from each end (assuming equal rates from each end). For example, the size of the MBN treated molecules at 6 hrs is ∼280 kb (versus ∼300 kb at the beginning), corresponding to 10 kb resection/DSB end/6 hours.

Using these PFGE conditions, the retardation in mobility of the resected, γ−linearized chromosomes appears to plateau at ∼1 hr (compare 1 and 6 hr in Figure 4B left side). The PFGE-shift of radiation broken molecules observed at early times (10–15 min) corresponds to at most hundreds of bases (not detectable as a change in linear size after MBN treatment). A plateau for mobility shift was also found for resection at HO-induced DSBs (Figure 3B) where resection occurs at only one end of each broken chromosome III fragment.

Thus, PFGE-shift provides a robust assay for detecting early events in resection at IR-induced random DSBs in *rad52* and *rad51* mutants (Figure 5B). In addition it provides an estimate of the proportion of molecules resected. These observations of PFGE-shift are consistent with the HO-induced DSB results (Figure 3B), the resected telomeres in *cdc13-1* cells (Figure 3A), and the shift in apparent sizes of γ−fragmented chromosomes (Figure 2B). Although suggested, there has been no clear direct evidence that single strand ends could affect mobility [31],[32].

For WT cells there is also PFGE-shift in broken Chr III molecules within 10 to 15 minutes postirradiation incubation, and the plateau in the shift (apparent size ∼430 kb) is reached for most molecules by ∼1 hr (Figure 4C). However, unlike for the *rad52* mutant, within the next hour the amount of unit-sized linear DNA is reduced, consistent with repair in WT cells. As expected, the MBN treatment of the shifted DNA (Figure 4D) led to a reduction in size demonstrating that the apparent increased molecular weight in Figure 4C was due to resected ends. The small reduction in size after MBN treatment is consistent with the ∼1.5 to 2 kb per hour resection rate observed for *rad52* over 6 hours. (In Figure 4D the chromosomes were separated under different PFGE conditions from those in Figure 4C in order to better assess the effect of MBN: see Materials and Methods.) Since substantial restitution is detected in WT cells at this time, repair of radiation induced DSBs is accomplished with single strand tails that are at most a few kb.

Unlike the situation for *rad52*, a second pair of shifted bands appeared with postirradiation incubation of WT cells beginning at ∼30 minutes. These two bands migrate at positions corresponding to ∼600 and ∼730 kb that are approximately double the size of the unresected and resected broken Chr III (Figure 4C; this region is not shown in Figure 4D because Chr III is obscured by the Chr II probe, *LEU2*). Given the central role for *RAD52* in recombination and DSB repair, these large molecules are likely to be products of recombination between sister chromatids and are discussed below.

### Efficient resection at random and unique DSBs depends on *RAD50*

**Figure 6:**
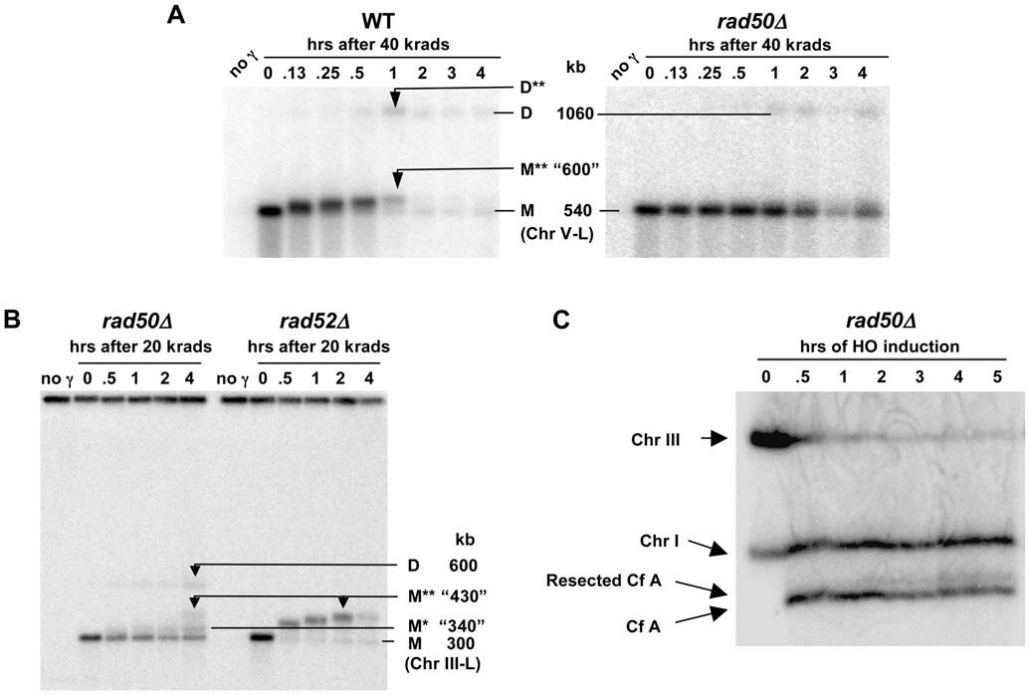
The PFGE-shift associated with a random γ-induced single DSB after low doses or HO-endonuclease produced DSBs. (A) PFGE-shift associated with a single radiation-induced DSB in circular Chr V in WT but not *rad50Δ* cells. Nocodazole arrested G2/M cells containing a circular chromosome V were irradiated with 40 krads and returned to YPDA media. Plugs were prepared at the indicated times and run on PFGE (TAFE). Southern transfers were hybridized with a *MET6* probe specific for the 530 kb Chr V. Results are similar to those obtained using circular Chr III, except the resected form of the putative linear dimer band (D**) seen at ∼1080 krads in WT (left image, 1 hr) is not well-resolved from the unresected form, twice the molecular weight of the non-resected monomer band (M). PFGE-shift of the linearized monomer band in WT reaches a maximum at an apparent molecular weight of ∼“600” kb at 1 hr, after which the M** band mostly disappears due to repair and recircularization. In the *rad50Δ* strain the non-resected monomer band (M) persists for 4 hrs with only faint smearing above it as also seen in *rad50Δ* using the circular Chr III construct (Figure 5A). The putative linear dimer band (D) was also detected with *rad50Δ* cells. (B) Resection events at low dose suggest one- and two-ended events. Arrested *rad50Δ* and *rad52Δ* G2/M cells containing circular Chr III were irradiated with 20 krads and returned to YPDA media. Plugs were prepared at the indicated time points and run on PFGE using CHEF analysis (see Materials and Methods). Two PFGE-shift bands, M* (at 0.5 to 4 hrs) and M** (at 4 hrs) were detected. As suggested in the text, the M* band is consistent with linearized molecules that were resected at only one end of the DSB. The M** seen in *rad50Δ* at 4 hrs is at the same maximum shift position as the M** band typically seen in *rad52Δ* by 2 hrs (see right group of lanes) and is proposed to be due to molecules that were resected at both ends with 3′ single-strand tails long enough to cause maximum shift. The persistence of significant portions of non-resected and of partially resected molecules in *rad50Δ* but not in *rad52Δ* demonstrates that *RAD50* is required for the rapid and efficient initiation of resection at damaged ends in WT and *rad52Δ* cells. (C) Lack of resection at HO-induced DSB in *rad50Δ* cells is reflected by absence of PFGE-shift. Resection was analyzed in the nocodazole-arrested *rad50Δ* cells (MN108) using procedures similar to those described in Figure 3B. Unlike for WT (Figure 3B), there was very little PFGE-shift, even at 5 hr.

Events in the *rad50Δ* mutant after γ-linearization of circular Chr III differ dramatically from WT and the *rad51Δ* and *rad52Δ* mutants. Rather than a rapid accumulation of the shifted ∼430 kb band, there is a slight “smear” in only ∼20% of the molecules beginning at about 1 hr after an 80 krad dose (compare Figure 5A with Figures 4A and 4C). Similar results were found for another circular chromosome, Chr V (540 kb; Figure 6A) following a dose of 40 krad. (Because of the nearly two-fold increase in size, the dose was reduced two-fold to induce a comparable number of broken chromosomes.) As shown in Figure 5B, differences in genetic control of the appearance of shifted molecules are even more distinct using CHEF gel conditions (see Materials and Methods for TAFE vs CHEF procedures). Treatment of the plugs from the *rad50Δ* mutant with MBN decreases the slight smear of the linear Chr III band, indicating that the shifted material is due to resection (Figure 5C). Based on the persistence of a large portion of unshifted and, therefore, unresected 300 kb molecules from the *rad50Δ* cells, the MRX complex is concluded to be essential for the rapid initiation of resection of radiation induced DSBs in WT, *rad52* and *-51* G2/M arrested cells (also growing cells; data not shown).

Like the WT cells, an apparent double size linear molecule is generated in the *rad50Δ* mutant by 30 minutes after irradiation for both Chr III (Figures 5A and B) and Chr V (Figure 6A). There is also a faint double-size band observed with the *rad51* mutant that shifts upward with time after irradiation (Figure 5B), but none for *rad52*.

**Figure 7:**
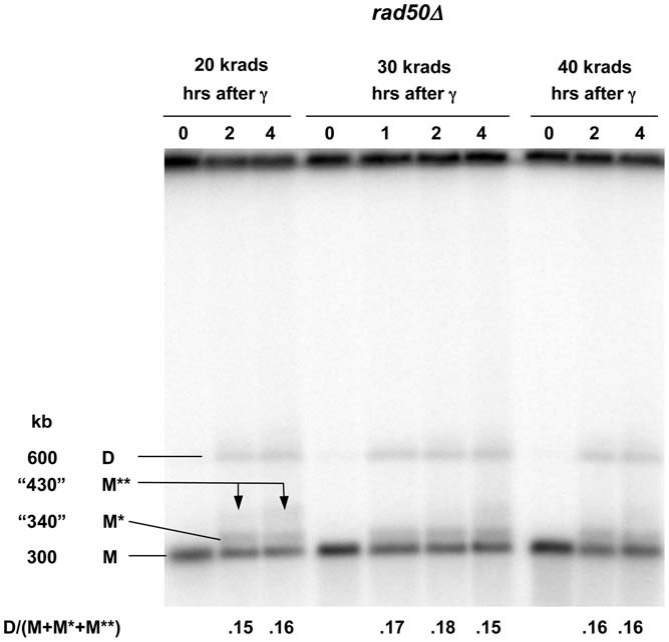
Relationship between dose and dimer formation in *rad50Δ*. Nocodazole arrested *rad50Δ* G2/M cells containing the circular Chr III were irradiated with 20, 30, and 40 krads and returned to YPDA media. Samples for PFGE were run on a CHEF (see Materials and Methods). The Southern transfer was hybridized to the Chr III-specific *CHA1* probe. The bands correspond to unresected monomer (M), putative one-end resected monomer (M*) and two-end resected monomer (M**), as well as putative dimer (D) (see Figure 5 B). Bands were quantitated using Kodak MI software and the ratio of total dimer DNA (D) to monomer DNA (M+M*+M**) for each time point is shown below each lane.

The difference in shifted molecules between the *rad50 vs* the *rad51* and *rad52* strains (Figure 5B) led us to examine events at an even lower dose (using the gel conditions of Figure 5B). For the *rad52* mutant most molecules exhibit PFGE-shift within 30 minutes after 20 krad and the apparent size is ∼430 kb by 1 hour, as described in Figure 6B. The pattern for the *rad50* mutant is quite different: ∼20% of the molecules exhibit an early small shift (∼340 kb) comparable to that seen at 80 krad. However, at later times molecules are detected that appear to be ∼430 kb, similar to the PFGE-shift found for the *rad52* mutant. Changes comparable to those with the circular Chr III in the *rad50* mutant were also observed with Chr V in the *mre11* mutant at 20 krad (Figure S3), demonstrating that the phenomenon is independent of chromosome and that a defect in either the *RAD50* or *MRE11* of the MRX complex can prevent efficient resection. These results are similar to our findings with bacteriophage lambda molecules that molecules with two resected ends exhibit a larger shift than molecules with one resected end (Figure S2B). We suggest that the fully shifted band observed in the *rad52* mutant is due to extensive resection at both ends of the DSB. Under the conditions of limited resection in the *rad50* and *mre11* mutants, the early shifted band would correspond to resection at only one of the broken ends. At lower doses where there are fewer DSB ends, the resection may also occur at the other broken end, eventually giving rise to the fully shifted molecules. At higher doses (*i.e.*, 40 and 80 krad), the opportunities for resection of the second end are reduced resulting in fewer fully shifted molecules (Figure 5B and 6A). (A dose-dependent decrease in the proposed two-end resected molecules is also seen with the *rad50* mutant, as described below in Figure 7.)

**Figure 8:**
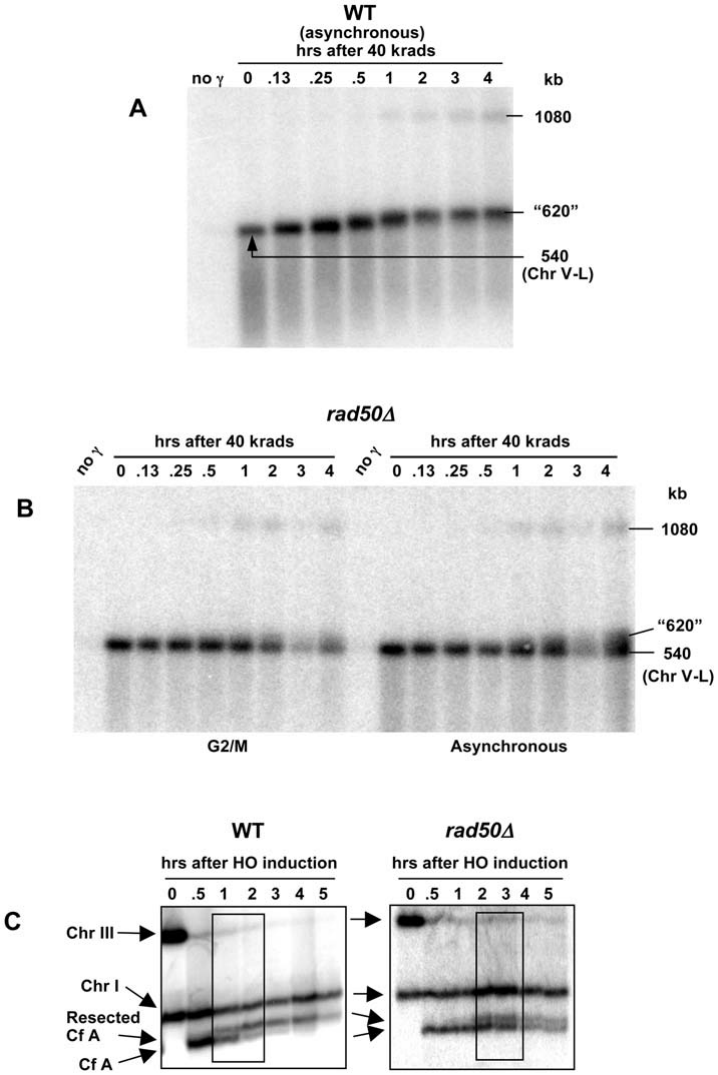
Resection at DSBs in logarithmically growing cells and role of *RAD50*. (A) Resection at a random DSB is rapid in asynchronous WT cells. Logarithmically growing WT cells containing the circular Chr V were irradiated (40 krads) and returned to growth conditions as described in Figure 1A and Materials and Methods. Samples were processed for PFGE and run on TAFE. The Chr V-specific *MET6* probe was used to hybridize the Southern blot. The kinetics of PFGE shift of nearly all molecules is similar to that observed for nocodazole-arrested cells (see Figure 6A). (B) Limited resection in G2/M and asynchronous *rad50Δ* cells. Logarithmically growing *rad50Δ* cells containing the circular Chr V were either arrested at G2/M with nocodazole (the G2/M image is reproduced from Figure 6A) or not arrested (right group of lanes) before irradiation (40 krads) and returned to growth in YPDA. Samples were processed for PFGE (TAFE) and a Southern transfer was hybridized to the Chr V specific *MET6* probe. Although more molecules experience Rad50-independent resection in asynchronous cells than in G2/M cells, the persistence of unshifted/unresected molecules as compared to WT (Figure 8A) indicates an important role for Rad50/MRX in initiation of resection even in asynchronous cells. (C) Resection of HO-cut chromosome fragments in asynchronous WT and *rad50Δ* cells as evidenced by PFGE-shift. The resection was analyzed in the asynchronous AM919 and MN108 cells (*rad50Δ*) using methods similar to those described in Figure 3B. The persistence of unresected “Cf A” molecules in *rad50Δ* indicates an important role for Rad50/MRX for initiation of resection. However, in asynchronous cells the requirement for Rad50 is not as great as in G2/M cells (Figure 6C).

We also examined the contribution of MRX to resection when there were only 2 DSBs (breaks in both sister chromatids) produced by HO-endonuclease in G2/M cells. As shown in Figure 6C (compare with Figure 3B), the efficient resection of the broken ends requires MRX and, as for the case of radiation-induced DSBs, there is a slight smearing with at most 5% of the molecules exhibiting a PFGE-shift. The lack of PFGE-shift in the *rad50Δ* strain was confirmed to correlate with a resection defect. Specifically, the efficiency of resection through the first 3 kb located proximal to DSB was assessed by loss of an EcoR1 restriction site located 3 kb proximal to the site of the break. While in the wild type strain more than 95% of molecules lost the sites by 5 hours, in *rad50Δ* strains less than 15% of the molecules lost the EcoR1 site. This is consistent with the absence of resection of HO-produced DSBs in G2-arrested *rad50Δ* cells but not asynchronous cells [33] (see Figure 8).

Exonuclease I which can function in replication and repair [34] appears to have a minor, possibly redundant role in γ-induced DSB repair (Figure 1A) and survival (Figure 1B), although overexpression can partially complement the radiation response and recombination defect of a *rad50Δ* mutant [19],[35]. As shown in S4A, the timing, extent of resection and disappearance of singly broken Chr III molecules due to repair in the *exo1* mutant was similar to that in G2 arrested WT cells, although resection appears be somewhat slower in the *exo1* mutant (circular Chr III in Figure S4B and circular Chr V, data not shown), suggesting a redundancy of systems available for resection and consistent with findings for HO-induced DSBs [16],[17].

Overall, these results suggest that regardless of which chromosome experiences DNA damage the initiation of resection in G2/M cells occurs in most molecules shortly after irradiation and is primarily dependent on MRX (also discussed below). In the MRX mutant, there is only a small capability for initiation of resection regardless of dose or whether DSBs are dirty (radiation-induced) or clean (HO-induced).

These findings extend to asynchronous cells. As shown in Figure 8, the WT strain also exhibited rapid shift of nearly all linearized Chr V molecules following a dose of 40 krad. This contrasts with the *rad50* mutant where even at 2 hr after irradiation most molecules do not exhibit a significant shift in mobility. Similarly, resection of the DSBs produced by HO in the asynchronous cells is also influenced by *RAD50*. However, a distinctly larger frequency of molecules are resected as compared to the G2/M cells, consistent with other measurements of resection at HO-induced DSBs in log cells [33].

### Recombination that is independent of *RAD50-* and *EXO1-*associated resection

During postirradiation incubation of WT, *rad50*, *mre11* and *exo1* strains a band is detected on Southerns within 30–60 minutes after irradiation [WT, Figure 4C; *rad50*, Figure 5A & 5B; mre11, Figure S3; *exo1* (data not shown)] that appears twice the unit length of the linearized chromosome. Since this double-size chromosome, which we refer to as a dimer, is not seen in *rad52* (Figure 4A and 5B), we conclude that it is the product of recombination (see Discussion and Figure S5). Unlike the *rad50* mutant, in the WT there are two recombinant bands (600 and “730” kb) that likely reflect a greater opportunity for resection (Figure 4C). The frequencies of recombinant molecules in the *rad50* mutant at 1 hr may be somewhat less than those found for the WT cells.

These results suggest that radiation-induced DSBs can efficiently generate MRX independent recombinants involving at most only a few hundred bases of resected DNA. This is consistent with observations that spontaneous [36] and site-specific DSB induced recombination [37] could occur in *rad50* mutants, including oligonucleotide mediated recombinational repair of a DSB [38]. The induction of recombinants is chromosome independent and a similar frequency was found after 80 krad to *rad50* cells containing the ∼300 kb Chr III (Figure 5A) or a lower dose (40 krad) to cells with the 540 kb Chr V (Figure 6A).

To address whether recombinants involve single- or multihit events, G2/M cells containing the circular Chr III were irradiated with 20 (also see Figure 6B), 30 and 40 krad and incubated for 2 and 4 hr. As shown in Figure 7, recombinants were clearly present at 2 hr (as well as at the 1 hr time point examined after a 30 krad dose) and the amount did not change in the next 2 hours, consistent with the above observations. Importantly, over this range the ratio of the signal in dimer (D) vs monomer (M+M*+M**) is comparable (∼15%; Figures 6B and 7); at 80 krad the amount is ∼25% (Figure 5B) [note that the probe intensity per molecule is expected to be twice more for dimer vs monomer]. This lack of dose dependence for appearance of recombinant molecules implicates single hit events in pairs of sisters (see Discussion and Figure S5) in the *rad50* strain under largely resection-independent conditions. However, the findings do not exclude a contribution of a single, full crossover when each sister contains a single DSB.

A faint recombinant band is also detected with the *rad51* mutant (Figure 5B). [Similar to the WT strain (Figure 4C), there is a PFGE-shift of the recombinant band which would result from resection of the dimer]. However, the appearance of the band is delayed and at a much lower level: ∼5% at 2 hr vs 15% for the *rad50* strains. Since there is no recombinant band in the *rad52* mutant and since resection is efficient in the *rad51* strain, this band might arise by single-strand annealing between overlapping resected regions at DSBs in pairs of sister chromatids.

## Discussion

### Detection of resection events using PFGE-shift

We have established that resection at random, synchronously induced dirty DSBs is primarily a *RAD50* and *MRE11* (MRX) dependent process generating up to a few kb of single-strand DNA prior to chromosomal restitution. These findings were facilitated by development of a robust system, based on PFGE-shift, for the efficient detection of resection at the ends of large molecules. Because DSBs are induced nearly instantaneously, radiation provides an opportunity for precise determination of early events in DSB processing, as compared to enzyme-induced DSBs. The resection-related PFGE-shift can be detected over a broad range of chromosome sizes, from tens of kb (lambda DNA) to large chromosomes (>800 kb Chr II) with resected telomeres. Surprisingly, the shift during postirradiation incubation appears even if the resected tail is a few hundred bases, as found in WT and *rad52* cells at 7.5 min after IR (based on resection in *rad52* cells over a 3 hr time period, as described in Figure 4B). The PFGE-shift system can also be used to estimate the number of molecules resected and may augment recently developed approaches that address resection at meiotic DSBs [39],[40].

### Resection, DSB repair, and implications

The resection detected by PFGE-shift occurs shortly after IR of WT cells at G2/M, with about 50% of singly broken molecules exhibiting a shift at 15 min after 40 or 80 krad (Figure 6A and 4D, respectively). Although most molecules undergo resection by 30 minutes, significant chromosomal restitution is not seen until 60 min after a dose of 80 krad (∼140 DSBs/cell) even for smaller chromosomes that experience less than an average of 1 DSB (data not shown). Most DSBs are repaired by 2 hr. (∼80%) based on the reappearance of chromosome bands (*i.e.*, Figure 1 and data not shown). The rapid initiation of resection may provide the programmed response needed for cell cycle arrest and also for induction of genes required for recombinational repair, such as *RAD51* [41] (also see Review [42]).

The resection associated with recombinational repair of radiation induced DSBs appears greater than the ∼1 kb involved in HO-induced recombination at MAT [37]. However, the amount of resection actually needed for recombination might be less. For example, only 50 nucleotides homology across a break is required for repair by a complimentary oligonucleotide [43]. The additional resection observed in these cases might provide an opportunity for loading sufficient Rad51 molecules to assure productive strand invasion and possibly induction of repair components (such as Rad51, [41].

There are several implications for the resected DNA following irradiation, including stress on nucleotide pools. At 1 to 2 kb per resected end and ∼140 DSBs per G2/M cell, about 300–600 kb of single-strand DNA would be generated in the first hour after irradiation, corresponding to ∼1–3% of the genome. Also, single strand DNA is particularly vulnerable to mutagenesis, suggesting a large window of genomic risk during DSB associated resection [44]. Furthermore, radiation-induced DSBs within TY elements are efficient at generating chromosome aberrations via TYs across the genome, even though there may be a sister chromatid available for repair [27]. The significant amount of end-resection would enable the large number of unbroken TYs in the genome to compete with sister chromatids for recombinational interactions. Since resection is limited to a few kb, it is unlikely that radiation would lead to chromosomal rearrangements via annealing between exposed repeat elements [32] generated at DSBs external to TYs.

The slowness of postirradiation resection (<0.5 to 1 base/sec in WT and a *rad52* mutant; Figures 4D and B, respectively) is similar to that for a single, HO-induced break under very different growth conditions ([30], and data not shown). It is interesting that it takes somewhat longer for the appearance of resected molecules following induction of a single pair of unique HO-induced DSBs in sister chromatids than for resection when there are over 100 DSBs per cell (Figures 3B and 6A), suggesting that dirty ends are not an impediment to the initiation of resection. Among the various reasons for the difference might be competition between resection and endjoining for HO-induced breaks [4],[17], only one sister chromatid being broken at the same location following γ radiation, and/or the systems for resection are less accessible to a programmed broken region.

### Genetic control of resection and DSB repair

While the MRX complex and Exo1 are important players in 5′→3′ resection at DSB ends, these are the first experiments to address directly their relative contribution to resection at damaged DSB ends. Previous experiments which had followed the timing of events based on the generation of foci demonstrated a rapid appearance of Mre11 foci following a low radiation dose (4 krad; [11]) and subsequent persistence of Mre11 followed by the accumulation of single-strand binding protein RPA in S phase and G2/M arrested cells [22]. We establish that the actual initiation of resection of radiation-induced DSB ends in G2/M cells is primarily dependent on *RAD50* and *MRE11* (MRX). Similar findings were obtained with an asynchronous population of growing cells, although there may be somewhat less dependence on MRX (Figure 8). Loss of Exo1 had only a small impact on resection at radiation-induced DSBs (Figure S4B), which correlates with the high survival in *exo1Δ* mutants following IR exposure.

The actual function of MRX or Exo1 in resection or in repair of DSBs induced by radiation remains to be determined. Recently, Williams *et al.* [45] proposed that MRX binds to both ends of a DSB and provides the opportunity for MRX itself or other redundant nucleases to carry out resection in a coordinated manner. Our *in vivo* results with the WT vs *rad50Δ* mutant appear to be consistent with that proposal. Within 30 minutes after 80 krad to WT, *rad52* and *rad51* strains, most linearized molecules are detected in a well-defined ∼430 kb PFGE-shift band. This contrasts with the *rad50Δ* mutant where the relatively few molecules that are resected after 80 krad exhibit limited PFGE shift (compare Figures 4C, 5A, and 5B). For lower doses (20 and 30 krad, see Figure 6B and Figure 7) to the *rad50Δ* mutant there is a similar pattern within the first two hours after radiation. However, by four hours, a much slower moving ∼430 kb PFGE-shift band is also observed which is comparable to that found in the WT and *rad50Δ* cells. We propose, based on the dramatic difference in PFGE for one-end vs two-end resected λ DNA molecules (Figure S2B), that the initial slow-moving band may be due to one-end resection, possibly by Exo1. The even slower-moving band appearing at later times which is comparable to that seen with *rad52* and WT cells would be due to the opportunity for resection at both ends of the linearized circular molecule with time after irradiation. Based on this interpretation, the MRX would assure coordinated resection at both ends of a DSB and efficient recombinational repair, possibly preventing half-crossovers (discussed below; see Figure S5). Since two distinct bands are detected with time after induction of a DSB, the results cannot be simply explained as being due to increasing time to carry out resection. The observation that both MRX and Exo1 ([46] (Nakai and Resnick, unpublished) are largely responsible for preventing a single DSB from transitioning to a cytologically detectable chromosome break supports the view that tethering DSB ends might influence opportunities for resection.

Additional proteins may also prove important in resection at γ-induced DSBs such as Sae2, an endonuclease that associates with MRX [47], the helicase Sgs1 implicated in recombination and repair of DNA damage [48],[49], the combination of Sgs1 and Dna2 [17], as well as Pso2 [50] which have little direct impact on radiation resistance. Recently, Huertas *et al.*, [15] demonstrated a cell-cycle dependent role in DSB processing for Sae2, an MRX interacting protein that has endonuclease activity and can act at inverted repeats [21]. The *sae2* mutants have a small increase in IR sensitivity (in terms of dose modifying factor) and reduced appearance of recombination proteins based on imaging analyses [15] indicating an early role for Sae2 in resection at damaged DSB ends, and this is currently under investigation (Westmoreland and Resnick, unpublished).

While the MRX complex is essential for efficient initiation of resection at an HO-break in nodocazole arrested G2/M cells it has less of a role in growing cells ([16],[17] and references therein) where resection at an HO-break can be accomplished in the absence of MRX through redundant activities of Exo1 or the Sgs1 helicase acting in concert with Dna2. The reason for this difference remains to be determined; however, it is possible that Sgs1/Dna2 activities associated with replication may partly obviate the role of MRX in initiation of resection.

### Recombination and resection

Recombination pathways account for nearly all repair of IR-induced DSBs in yeast and MRX is a key component (Figure 2A). We found no evidence of repair via endjoining mechanisms in G2/M arrested cells although an endjoining deficiency increases sensitivity of *rad52* cells to radiation [51]. The present results strongly support an important role for MRX mediated resection in the eventual DSB recombinational repair, although additional MRX activities may impact recombinational repair including tethering of ends and checkpoint signaling [52]. MRX, but not Exo1, appears to be required for the efficient recombinational repair of radiation-induced DSBs in G2/M cells (as well as growing cells), which is consistent with the much larger role that MRX plays in resection of DSBs (*e.g.* Figure 4C vs 5A and S3; also Figure S4 for *exo1*).

While the MRX mediated resection of radiation induced DSBs is required for recombinational repair, the circular chromosomes provide a unique opportunity to address another facet of DSB induced recombination. In WT cells, there was a rapid appearance of linear dimer molecules formed between sister chromatids and subsequent disappearance (presumably a result of completed repair) that was *RAD52* dependent. Surprisingly, dimer molecules were also formed at early times in the *rad50* strain, suggesting that at least some steps in recombination in the G2/M cells could occur with, at most, limited resection. It will be interesting to identify the components of that process and their contribution to damage-induced crossovers between sister chromatids and homologous chromosomes [53]. If these *rad50* independent dimers require generation of single strand regions, an alternative intermediate might involve DNA unwinding to create ends that could lead to strand invasion. A 3′to 5′ Rec Q helicase such as SGS1 (homologue of Werner and Bloom helicases) [54] or SRS2 [55] might provide this function, possibly after a very small amount of resection as found for its role in resection at a clean HO-induced DSB [16],[17]. It is unlikely that the recombinants are due to single strand annealing, especially at low doses, since the breaks would have to be in the same vicinity on the sister chromatids; the recombinants are actually detected shortly after irradiation when there would be at most only a small amount of resection, especially in the *rad50* mutant. It is also possible that recombinant molecules could be generated through a mechanism of break-induced replication (BIR), but that is a slow process [56] (contrary to what we observe) and furthermore would not be expected to generate the linear, double-unit molecules that we see.

The late appearance of dimers in the *rad51* mutant (Figure 5B) might be attributed to single stand annealing. Single-strand annealing between broken molecules has been found for interchromosomal chromosome aberrations originating within TY elements in yeast following IR [27]. Extensive resection and annealing has also been suggested as a mechanism for restoration of the genome of *Dinococcus radiodurans* following exposure to high radiation doses [57],[58].

We have asked whether the full-length linear dimers result from one or two DSBs in sister chromatids as described in Figure S5. For two-hits, each sister chromatid in a cell has a single DSB (or two hits in one sister and no hits in the other sister) and one of the breaks leads to a reciprocal exchange. In a one-hit model one sister is broken and the other is not, in which case the initiation of recombinational repair involving one of the ends will lead to a transient half crossover in the WT. Processing of only one end in the *rad50* mutants at early times (as suggested above) may preclude the opportunity for full crossovers. Based on the actual observed frequencies and predicted incidence of single DSB or two DSBs in a pair of molecules (analysis not shown) we are unable to distinguish between the two models over the 20 to 80 krad range examined for the *rad50* mutant, although the results best accommodate a single hit model in the range of 20 to 40 krad.

In WT cells, the recombined dimer molecules (that also appear to undergo resection) are lost during the time when there is significant restitution of full-length linear chromosomes (*e. g.*, Figure 4C). Based on irradiation of the DNA plugs and PFGE analysis, the dimer molecules are repaired and generate circular dimer chromosomes (Westmoreland and Resnick, unpublished). The appearance of circular dimer molecules following IR treatment was previously reported by Game *et al.* [53]. The present results do not address the extent to which crossovers induced by radiation in WT cells are dependent on MRX determined resection. However, if the appearance of linear dimers is an indicator, the incidence does not differ dramatically between WT and *rad50* mutants. Possibly, the MRX mediated resection assures an efficient gene conversion type of repair.

## Materials and Methods

### Yeast strains

All strains used in this study were haploids. The following two strains used for HO-endonuclease induced DSBs were derived from strain EI515 [59]: AM919, *MATa ade1 ura3-52 leu2-3,112 THR4 lys5 Chr III::URA3 hml*Δ*::ADE1 hmr*Δ*::ADE1 ade3::GAL::HO* [32]; MLN108, a *rad50Δ* derivative of EI515 [60].

All other yeast strains used in this study were derived from ALE100, described in [61] and [62]. Construction of DAG760 containing the *cdc13-1 ts* mutation (Figure 3A), is described in [44]. All strains containing circular chromosome III were derived from two isogenic strains MWJ49 and MWJ50 [26]. These strains have two copies of the *LEU2* gene, one in chromosome III (original location) and the other copy in the vicinity of *LYS2* in chromosome II. This allows detection of both chromosomes in Southern analysis with a Leu2 probe and is used in one of two methods to quantitate DSB induction efficiency (see Figure S1B). The circular Chromosome V was made in ALE100 using the break-mediated *delitto perfetto* method [63] creating strain JW1773. In short, a *GAL1*-I-*Sce*I core cassette was inserted 34 kb from the left telomere, immediately centromeric to the *CAN1* gene. A DSB was induced by galactose at the I-*Sce*I site in the cassette, and an integrative recombinant oligonucleotide (containing sequences homologous to both the right side of the I-*Sce*I break and also to a location 10.6 kb from the right telomere of Chromosome V) was used to circularize the Chromosome V to create strain JW1773. Strains with gene deletions were created in MWJ49, MWJ50, and JW1773 by replacement of the relevant ORFs with the G418 (*kanMX4*) cassette as described [64]. Double-deletion strains were generated by switching the G418 resistance marker in the first deletion to hygromycin (*HphMX4*) followed by replacement of the second ORF using the *kanMX4* cassette. All construction steps were verified by PCR and phenotype.

### Nocodazole arrest, gamma irradiation, and post-irradiation incubation

The details on nocodazole arrest, flow cytometry and gamma irradiation have been described [27]. Briefly, nocodazole (20 µg/ml final concentration) was added to cells growing logarithmically at 30°C in YPDA media (1% yeast extract, 2% Bacto-Peptone, 2% dextrose, 60 µg/ml adenine sulfate). G2 arrest was monitored by cell morphology and verified by flow cytometry. Cells were harvested by centrifugation, washed and resuspended in ice-cold sterile water. The cell suspensions were kept on ice while being irradiated in a ^137^Cs irradiator (J. L. Shepherd Model 431) at a dose rate of 2.3 krads per minute. Irradiated cells were harvested by centrifugation and resuspended in YPDA at 30°C with nocodazole for post-irradiation incubation.

### HO-endonuclease–induced DSB and detection of resection

Cells (EI515 and MLN108) were grown overnight in YPDA and transferred to YEP lactate (1% yeast extract, 2% peptone, 3.7% lactic acid, pH 5.5, 60 µg/ml adenine sulfate) and grown overnight. HO was induced when cell density was 1×10^7^ cells/ml by adding 2% galactose, final concentration. Greater than 95% of cells experienced a DSB within 1 hr after adding galactose. The time course of resection was determined as described previously [56]. In experiments with G2/M arrested cells, nocodazole (final concentration 0.015 mg/ml) was added 3 hours prior to addition of galactose. For PFGE (Figure 3B and 6C), chromosomal plugs were prepared using the CHEF genomic DNA plug kit (Bio-Rad, Hercules, CA). PFGE was performed using genomic DNA embedded in plugs of 1% agarose. The DNA was subsequently examined by Southern analysis, and blots were probed with *ADE1*-specific fragment labeled with P^32^. Blots were analyzed using a Molecular Dynamics PhosphorImager. To account for variation in DNA loading, intensities of the bands corresponding to the intact chromosome III, or to the various forms of cut fragments resulting from breakage of chromosome III by HO were normalized to intensities of the bands corresponding to chromosome I, which also hybridizes to the *ADE1*-specific probe. The 5′ processing at a DSB end was assessed using the methods described in [17]. In particular, the DNA was digested using EcoR1. To detect 5′ resection beyond the EcoR1 site the radiolabeled probe specific to the *PHO87* gene was used. Quantities of DNA loaded on gels for each time point were normalized using *RAD54-*specific DNA probe.

### PFGE procedures

Separation of chromosomes was accomplished with Transverse Alternating Field Electrophoresis (TAFE) and Contour-clamped Homogeneous Electric Field (CHEF) systems. A Gene Line II apparatus (Beckman Instruments, Fullerton, CA ) was used for TAFE and a CHEF Mapper XA system (Bio-Rad, Hercules, CA) was used for CHEF. In the early part of the study, TAFE was used for PFGE gels due to the sharper bands on stained gels (Figures 1A, 2B, 4A, 4C, 5A, 6A, S1B, S2A, S2B, S4A and S4B). Plugs were prepared according to manufacturer specifications. CHEF gels were used in the mung bean nuclease experiments (Figures 4B, 4D and 5C) where running conditions were changed to enable better discrimination of molecules with digested single strand tails (under these conditions, there was less PFGE shift). CHEF gels were also used in later experiments (Figures 2A, 5B, 6B, 7 and S3) because they enabled better characterization of partially shifted bands on Southerns. For the CHEF experiments, a modified plug preparation technique (adapted from Juan Lucas Argueso, personal communication) was used. Plugs were prepared in 0.5% LE agarose (Seakem, Rockland, ME) using 6×10^7^ G2-arrested cells per 100 ul plug. They were cut to a thickness of ∼2 mm and loaded in the bottom of a preparative well so that all the DNA migrated very close to the bottom surface of the CHEF gel. This minimized the distance that the DNA had to travel through the thickness of the gel during Southern transfer and also reduced the band smearing that occurs in the gel itself during CHEF electrophoresis. Specific program parameters are available upon request. Lambda DNA ladders were used as molecular weight standards.

### Mung bean nuclease digestion of DNA in PFGE plugs

Single-stranded overhangs at the sites of DSBs resulting from resection were removed by mung bean nuclease resulting in DSBs with blunt ends and a change in the PFGE mobility (described in the Results). Mung bean nuclease with 10× reaction buffer was purchased from Promega (Madison, WI). For digestion of yeast plugs, a 50 ul plug slice was equilibrated 3 times for 20 minutes at room temperature in 150 ml of TE (10 mM Tris, pH 7.4, 1 mM EDTA), followed by 20 minute incubation at room temperature with 40 units/ml of mung bean nuclease (controls were incubated in reaction buffer without enzyme) and finally equilibration on ice in 50 mM EDTA to stop the reaction. (In early experiments proteinase k treatment to destroy the enzyme was found to have no effect on the PFGE results; therefore, its use was later omitted.) The molecular weights associated with bands were calculated using MI (version 4.0) software from Eastman Kodak (Rochester, NY) by comparing with positions in length marker bands (lambda DNA ladder; New England Biolabs, Beverly, MA).

### Southern transfer and hybridization

Neutral Southern blots, probe preparations, ^32^P labeling and hybridizations were carried out as previously described [26]. Primers used for PCR amplification of genomic DNA to be used in the preparation of probes are listed in Table S1.

### Quantitation of DSBs

Two methods were used to calculate DSB induction efficiency using PFGE. Both methods assume that DSB induction is random throughout the genome. For both methods, Kodak MI software (version 4.0) was used to quantify bands used in the analyses.

#### Stained gel, multiple-band method

This method requires neither Southern analysis nor the presence of a circular chromosome. To quantify DSBs in irradiated samples, pulsed-field gels were stained with SybrGold (Invitrogen, San Diego, CA) and photographed using a GelLogic200 imaging system (Eastman Kodak, Rochester, NY). Bands were measured using Kodak MI software (version 4.0) and the data were exported into Microsoft Excel (version 11.5.3) for further manipulations to determine DSBs. Details on the analysis to determine DSBs are available upon request, and an example of the calculations is shown in Figure S1A. Briefly, for each band corresponding to a complete unbroken chromosome Y, the fraction of chromosomes remaining unbroken (*F_ChrY_*) after a given dose is simply the net intensity of the band divided by the net intensity of the corresponding band in the 0 krad control lane. From the Poisson distribution, the average number of DSBs (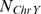) is given by the formula:
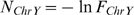
The values for *F_ChrY_* and *N_ChrY_* at a given dose (*i.e.*, each lane corresponds to a dose) are accurate to the extent that an equal amount of total DNA was loaded in the 0 krad lane as in the lanes with irradiated DNA. Plotting the experimentally determined values of *N* vs MW for each chromosome band from a given dose results in an approximate straight line whose slope is in units of DSBs/mb and is independent of the total amount of DNA loaded in each lane as long as enough DNA is loaded for accurate detection of the bands (Figure S1A). The experimentally determined values of the slope (DSBs per mb) for a given dose are highly reproducible and an example is given in Figure S1A.

#### LEU2 probe method (two bands, single probe of Southern blot)

This is described in detail in [26] and in Figure S1B. A probe homologous to a sequence in the *LEU2* genes on both the linear chromosome II and a circular Chromosome III was hybridized to Southern blots of pulsed-field gels of plugs made from yeast that had been treated with 0 to 80 krads. The number of DSBs per chromosome III at each dose was calculated by taking the relative intensity of the peak of singly broken (*i.e.*, linearized by gamma, but full length) circular chromosome III to that of unbroken chromosome II and applying the following formula:
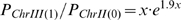
(*P_Chr III(1)_*) and (*P_Chr II(0)_*) are the probabilities of a single break in chromosome III and no breaks in chromosome II when the average number of DSBs in chromosome III is (*X*).

Over the range of 10 to 60 krads, there was very good proportionality of DSBs per chromosome with the dose used to induce DSBs (R^2^ = 0.9992 for the results shown in Figure S1B). Above 60 krads, the intensity of the chromosome II band was too low to be quantified.

### Enzymatic treatments of lambda DNA

Bacteriophage λ DNA, λ-exonuclease, and *E. coli* exonuclease I were purchased from New England Biolabs (NEB, Beverly, MA) and were used with supplied buffers according to manufacturer's instructions.

#### In vitro resection of λ DNA with λ exonuclease

λ DNA was digested at 37°C at a final concentration of 20 µg/ml DNA and 147 units/ml of λ-exonuclease. Reactions were stopped on ice with EDTA at a final concentration of 20 µg/ml and heat inactivated at 75°C for 10 minutes. Plugs for PFGE were prepared by mixing equal volumes of sample with a solution of molten 1% LE agarose (Seakem, Rockland, ME) in 200 mM EDTA at 45°C and subsequently transferring to a plug mold. After hardening at 4°C, the plugs were either treated with *E. coli* exonuclease I as described below to remove the 3′ single strand tails or treated for 2 hours with 1 mg/ml proteinase K (Invitrogen, San Diego, CA) and 1% sarcosyl at 37°C. Samples were stored at 4°C until run on PFGE.

#### Digestion of in vitro resected (λ exonuclease treated) λ DNA with *E. coli* exonuclease I to remove single strand 3′ tails

Plugs of resected λ DNA (∼50 µl volume) were equilibrated in 500 µl of TE (10 mM Tris, pH 8.0, 1 mM EDTA), and then incubated in 200 µl of 1.25× reaction buffer (supplied by NEB) with 100 units of exonuclease I at 37°C for 30 minutes. Immediately after the exonuclease I incubation, plugs were transferred to 1 ml 100 mM EDTA containing 1 mg/ml proteinase K and 1% sarcosyl and incubated for 2 hours at 37°C. Samples were stored at 4°C until run on PFGE.

## Supporting Information

Figure S1 Two approaches to estimating DSB induction. (A) Stained gel, multiple band method. As described in Materials and Methods, for each band corresponding to chromosome Y, the fraction of chromosomes remaining unbroken (*F_ChrY_*) after a given dose is simply the intensity of the Chr Y irradiated band divided by the intensity of the corresponding band in the 0 krad control lane. Therefore, the average number of DSBs, *N_ChrY_*, per molecule for any chromosome Y is equal to −ln *F_ChrY_*. The values for *F_ChrY_* and *N_ChrY_* at a given dose (i.e., each lane corresponds to a dose) are expected to be accurate to the extent that equal amounts of total DNA were loaded in the 0 krad lane as in the lanes with irradiated DNA. Plotting the experimentally determined values of *N_ChrY_* vs MW for a given dose is expected to result in straight line whose slope is in units of DSBs/mb. Presented in (A) is an example of PFGE band intensities (SybrGold) for different chromosomes from cells receiving no irradiation or 30 krad, along with *F_ChrY_* and *N_ChrY_* values which are shown in the table in (A). These values are plotted against MW and yield a slope of 2.2 DSBs/mb at 30 krad. In the following discussion, we establish that the slope is actually independent of the total amount of DNA loaded in each lane even though individual *N_ChrY_* values are influenced by relative amounts of DNA between the lanes. *N_ChrY_* = Fraction of unbroken ChrY remaining after gamma (irradiated peak/unirradiated peak) *N_ChrY_* = number of DSBs per ChrY = −ln *F_ChrY_*. This applies to situations where the amounts of DNA in the irradiated and unirradiated lanes are equal. Now consider that there is a difference (factor *R*) in loading between the irradiated and unirradiated lanes. This would affect the amount of material in all bands to the same extent. Therefore, the measured value *F′_ChrY_* is really *R* times the true value of *F_ChrY_* (i.e., when there is equal loading), and the measured value *N′_ChrY_* = −lnR *F_ChrY_* = −(lnR+ln *F_ChrY_*). Since the value *R* is the same for all bands in the lane with irradiated DNA, all measured values of *N′_ChrY_* obtained for different chromosomes Y will differ from the true value of *N_ChrY_* by the same quantity (−ln *R*) and the resulting slope (DSBs per mb) would be the same as if there was no loading difference between lanes. (B) Estimating DSB frequency using probe to common sequence in linear Chr II and circular Chr III. In the method described by Ma et al. [26], a *LEU2*-probe, which identifies Chr II and Chr III, is used in Southern hybridization of a pulsed-field gel. Circular chromosome III molecules remain trapped in the well. However, a single random break in circular chromosome III results in full-length linearized chromosome III molecules that can migrate out of the well and form a unique band (as indicated). For the linear chromosome II (Chr II), one or more breaks result in fragmentation and disappearance of material from the linear chromosome II band. The ratio between the amount of the remaining intact chromosome II band and the damaged-induced linearized chromosome III band is used to calculate the DSBs/G2 cell by the equation shown (see [26]; also Materials and Methods). The positions of linearized, singly broken chromosome III and unbroken, linear chromosome II are indicated. In this experiment, G2-arrested, WT haploid cells were irradiated with 80 krad and samples were run on PFGE (TAFE; see Materials and Methods), followed by Southern transfer and probing with a *LEU2* probe to identify chromosomes II and III. The number of DSBs per haploid G2 genome is shown beneath each lane.
(1.34 MB TIF)

Figure S2 PFGE-shift detected with λ DNAs. (A) PFGE-shift of resected λ DNA. λ DNA was resected with lambda exonuclease as described in Materials and Methods. Samples were stopped at the indicated time points by diluting in ice-cold EDTA solution, and plugs were prepared for PFGE (TAFE; see Materials and Methods). For the “0” time sample, a 48.5 kb lambda ladder formed due to annealing between the 12 base overhangs at the ends of full-length (unresected) lambda DNA molecules. The resection rate was determined by progressive disappearance of Hind III restriction sites. (B) PFGE-shift of one-end resected and two-end resected λ DNA fragments. One-end resections were created by first treating λ DNA with lambda exonuclease for the indicated times, then inactivating the lambda exonuclease at 65°C and finally restricting the resected λ DNA with PspOM I before running on PFGE (TAFE). For two-end resections, λ DNA was restricted with PspOM I before resection of the 38.4 and 10.1 kb PspOM I fragments. “Trimming” of two-end resected PspOM I fragments was done using *E. coli* exonuclease I to remove the 5′ tails.
(2.99 MB TIF)

Figure S3 Resection and recombination are comparable in *mre11* and *rad50* mutants. Arrested *mre11*, *rad50*, and *rad52* G2/M cells containing circular Chr V were irradiated with 20 krads and returned to YPDA media. Plugs were prepared from the indicated time points and run on PFGE (CHEF, see Materials and Methods). Presented is a Southern of the gel using a Chr V specific *MET6* probe. The results mirror those seen in Figure 6B with the circular Chr III following 20 krads exposure to the *rad50* and *rad52* mutants. Two PFGE-shift bands, M* (at 1 to 4 hrs) and M** (at 4 hrs), as well as a putative recombinant dimer band, D (at 0.5 to 4 hrs), were detected in both the *mre11* and *rad50* strains. In *rad52*, only the M** PFGE-shift band (proposed two-end resection of linearized circular Chr V) appeared, and this band appeared rapidly after the irradiated cells were returned to growth media. As discussed in the text, the M* band seen in *mre11* and *rad50* is likely to be composed of linearized circular Chr V molecules resected at only one end.
(3.64 MB TIF)

Figure S4 Role of *EXO1* in resection at random, radiation induced DSBs. (A) Resection at a random break is slower in *exo1Δ* strains. Logarithmically growing WT and *exo1Δ* cells containing circular Chr III were arrested at G2/M, irradiated with 80 krad, and returned to YPDA as described in Figure 1A and in Materials and Methods. Samples were processed and run on PFGE (TAFE, see Materials and Methods). A Southern transfer of the TAFE gel was hybridized to the Chr III-specific CHA1 probe. While most molecules exhibited a PFGE-shift, the resection rate in *exo1Δ* appears to be somewhat slower than in WT, but much greater than in *rad50*. Chromosomal repair in the *exo1* strain is comparable to that of the WT strain (Figure 1A). (B) Further comparison of resection in WT and *exo1Δ* strains. These images correspond to the images in Figure S4A except that the *exo1Δ* image (right) has been flipped horizontally to better reveal the slightly reduced PFGE shift at one hr in the *exo1Δ* strain vs the WT. Similar results were obtained in the *exo1Δ* strain containing a circular Chr V (data not shown). Although the difference in PFGE shift between the two strains at each time point is small, the actual processivity of resection could be affected as much as two-fold. Since most molecules from the *exo1Δ* time course exhibit PFGE shift by 1 hour, initiation of resection is not greatly affected by the absence of exonuclease 1.
(2.22 MB JPG)

Figure S5 Models describing the generation of linear dimer molecules from broken circular sister chromatid. (Figures are adapted from [65] in their study of double-strand break mediated recombination in meiosis.) (A) In the wild type cells, a single DSB in one of two sister chromatids can be repaired via resection, strand invasion, strand extension by DNA synthesis, reannealing with complementary resected end and resolvase resolution of a Holiday junction to generate a circular dimer. A temporary half crossover could occur through migration of the Holiday junction and resolvase activity (lower part of (A)). (B) In cells with little or no resection of at least one end of a DSB, opportunities for completion of recombinational repair of a single DSB are reduced. Very limited resection or helicase generation of short single strand regions might allow recombinational interactions, resulting in half crossovers. (C) Pairs of sister chromatids each with a DSB could result in a linear dimer if only one is repaired. At low doses the likelihood of a cell having a single DSB in each sister chromatid is low. With increased dose, a single DSB in each chromatid becomes more likely. However, this scenario (a single DSB in each sister chromatid or two breaks in one sister and none in the other) eventually becomes less likely at increasingly higher doses that induce multiple DSBs per chromatid.
(1.03 MB TIF)

Table S1 Primers used for generation of probes used in this study.
(0.18 MB TIF)
