# Supplementary figures and images for: RAD50 Is Required for Efficient Initiation of Resection and Recombinational Repair at Random, γ-Induced Double-Strand Break Ends
Source: PLoS Genet. 2009 Sep 18;5(9):e1000656. doi: 10.1371/journal.pgen.1000656 (PMC2734177; doi:10.1371/journal.pgen.1000656)

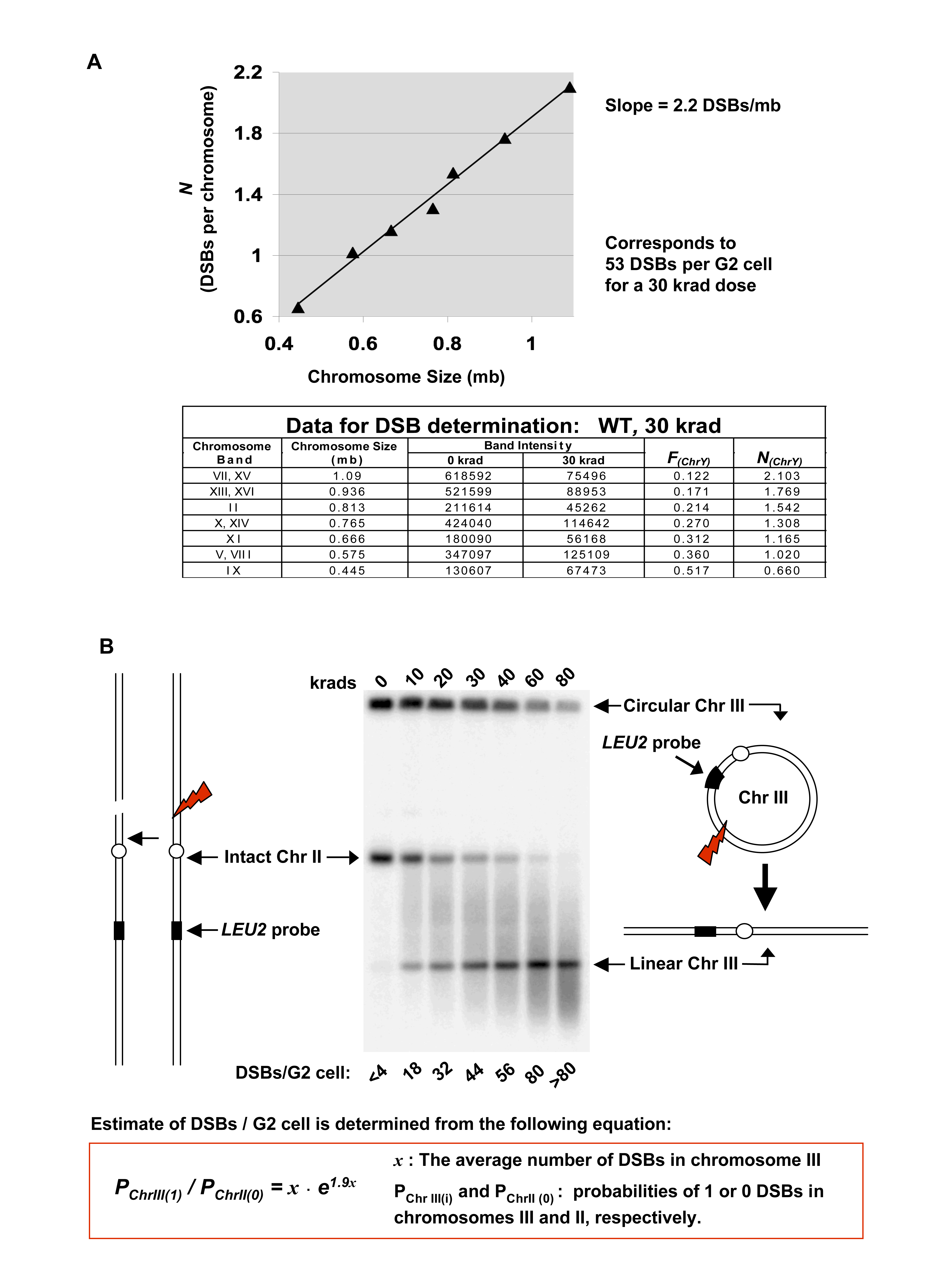

Supplement: Figure S1 — Two approaches to estimating DSB induction. (A) Stained gel, multiple band method. As described in Materials and Methods, for each band corresponding to chromosome Y, the fraction of chromosomes remaining unbroken (FChrY) after a given dose is simply the intensity of the Chr Y irradiated band divided by the intensity of the corresponding band in the 0 krad control lane. Therefore, the average number of DSBs, NChrY, per molecule for any chromosome Y is equal to −ln FChrY. The values for FChrY and NChrY at a given dose (i.e., each lane corresponds to a dose) are expected to be accurate to the extent that equal amounts of total DNA were loaded in the 0 krad lane as in the lanes with irradiated DNA. Plotting the experimentally determined values of NChrY vs MW for a given dose is expected to result in straight line whose slope is in units of DSBs/mb. Presented in (A) is an example of PFGE band intensities (SybrGold) for different chromosomes from cells receiving no irradiation or 30 krad, along with FChrY and NChrY values which are shown in the table in (A). These values are plotted against MW and yield a slope of 2.2 DSBs/mb at 30 krad. In the following discussion, we establish that the slope is actually independent of the total amount of DNA loaded in each lane even though individual NChrY values are influenced by relative amounts of DNA between the lanes. NChrY = Fraction of unbroken ChrY remaining after gamma (irradiated peak/unirradiated peak) NChrY = number of DSBs per ChrY = −ln FChrY. This applies to situations where the amounts of DNA in the irradiated and unirradiated lanes are equal. Now consider that there is a difference (factor R) in loading between the irradiated and unirradiated lanes. This would affect the amount of material in all bands to the same extent. Therefore, the measured value F′ChrY is really R times the true value of FChrY (i.e., when there is equal loading), and the measured value N′ChrY = −lnR FChrY = −(lnR+ln FChrY). Since the value R is t [file pgen.1000656.s001.tif]

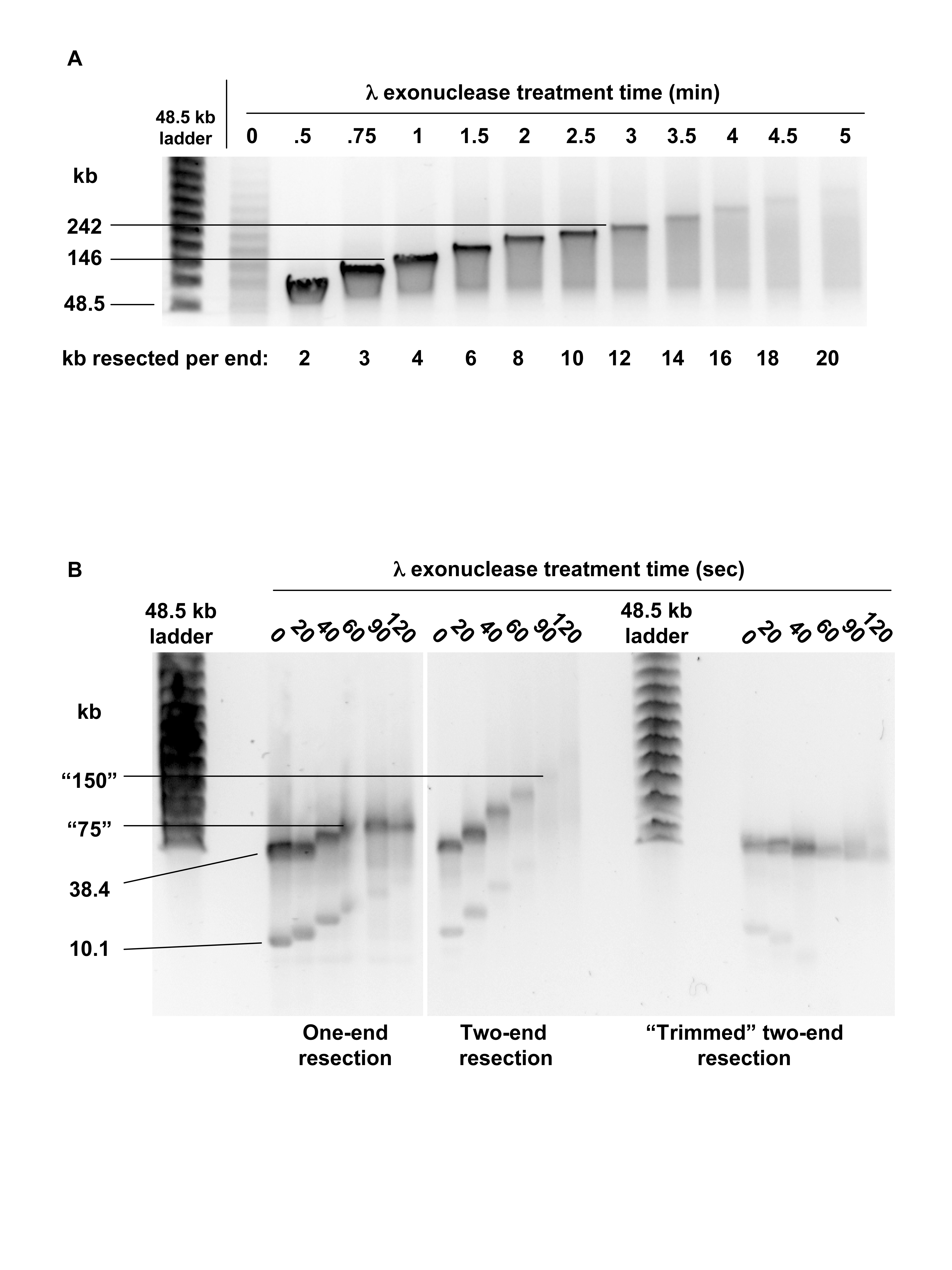

Supplement: Figure S2 — PFGE-shift detected with λ DNAs. (A) PFGE-shift of resected λ DNA. λ DNA was resected with lambda exonuclease as described in Materials and Methods. Samples were stopped at the indicated time points by diluting in ice-cold EDTA solution, and plugs were prepared for PFGE (TAFE; see Materials and Methods). For the “0” time sample, a 48.5 kb lambda ladder formed due to annealing between the 12 base overhangs at the ends of full-length (unresected) lambda DNA molecules. The resection rate was determined by progressive disappearance of Hind III restriction sites. (B) PFGE-shift of one-end resected and two-end resected λ DNA fragments. One-end resections were created by first treating λ DNA with lambda exonuclease for the indicated times, then inactivating the lambda exonuclease at 65°C and finally restricting the resected λ DNA with PspOM I before running on PFGE (TAFE). For two-end resections, λ DNA was restricted with PspOM I before resection of the 38.4 and 10.1 kb PspOM I fragments. “Trimming” of two-end resected PspOM I fragments was done using E. coli exonuclease I to remove the 5′ tails. (2.99 MB TIF) [file pgen.1000656.s002.tif]

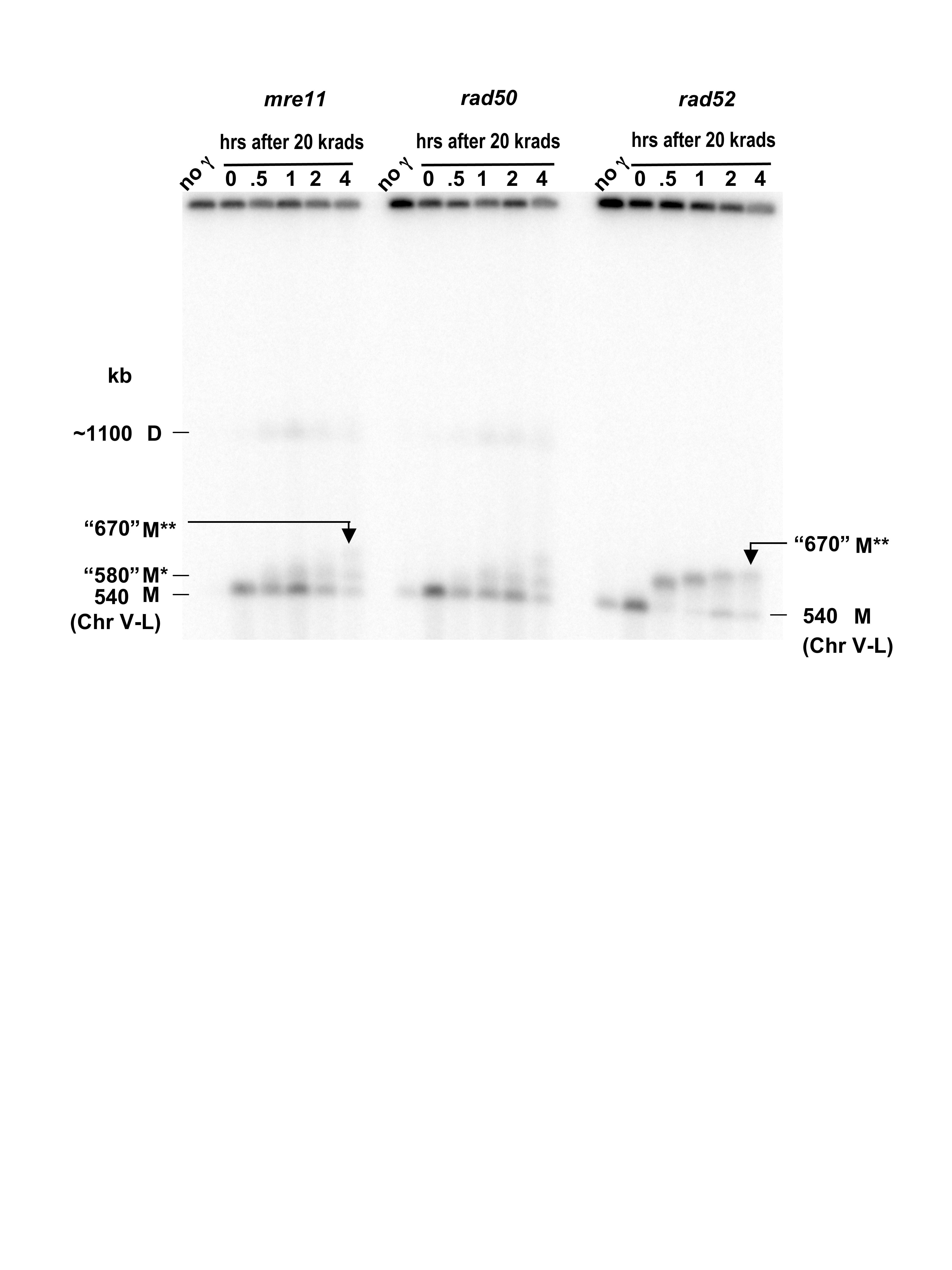

Supplement: Figure S3 — Resection and recombination are comparable in mre11 and rad50 mutants. Arrested mre11, rad50, and rad52 G2/M cells containing circular Chr V were irradiated with 20 krads and returned to YPDA media. Plugs were prepared from the indicated time points and run on PFGE (CHEF, see Materials and Methods). Presented is a Southern of the gel using a Chr V specific MET6 probe. The results mirror those seen in Figure 6B with the circular Chr III following 20 krads exposure to the rad50 and rad52 mutants. Two PFGE-shift bands, M* (at 1 to 4 hrs) and M** (at 4 hrs), as well as a putative recombinant dimer band, D (at 0.5 to 4 hrs), were detected in both the mre11 and rad50 strains. In rad52, only the M** PFGE-shift band (proposed two-end resection of linearized circular Chr V) appeared, and this band appeared rapidly after the irradiated cells were returned to growth media. As discussed in the text, the M* band seen in mre11 and rad50 is likely to be composed of linearized circular Chr V molecules resected at only one end. (3.64 MB TIF) [file pgen.1000656.s003.tif]

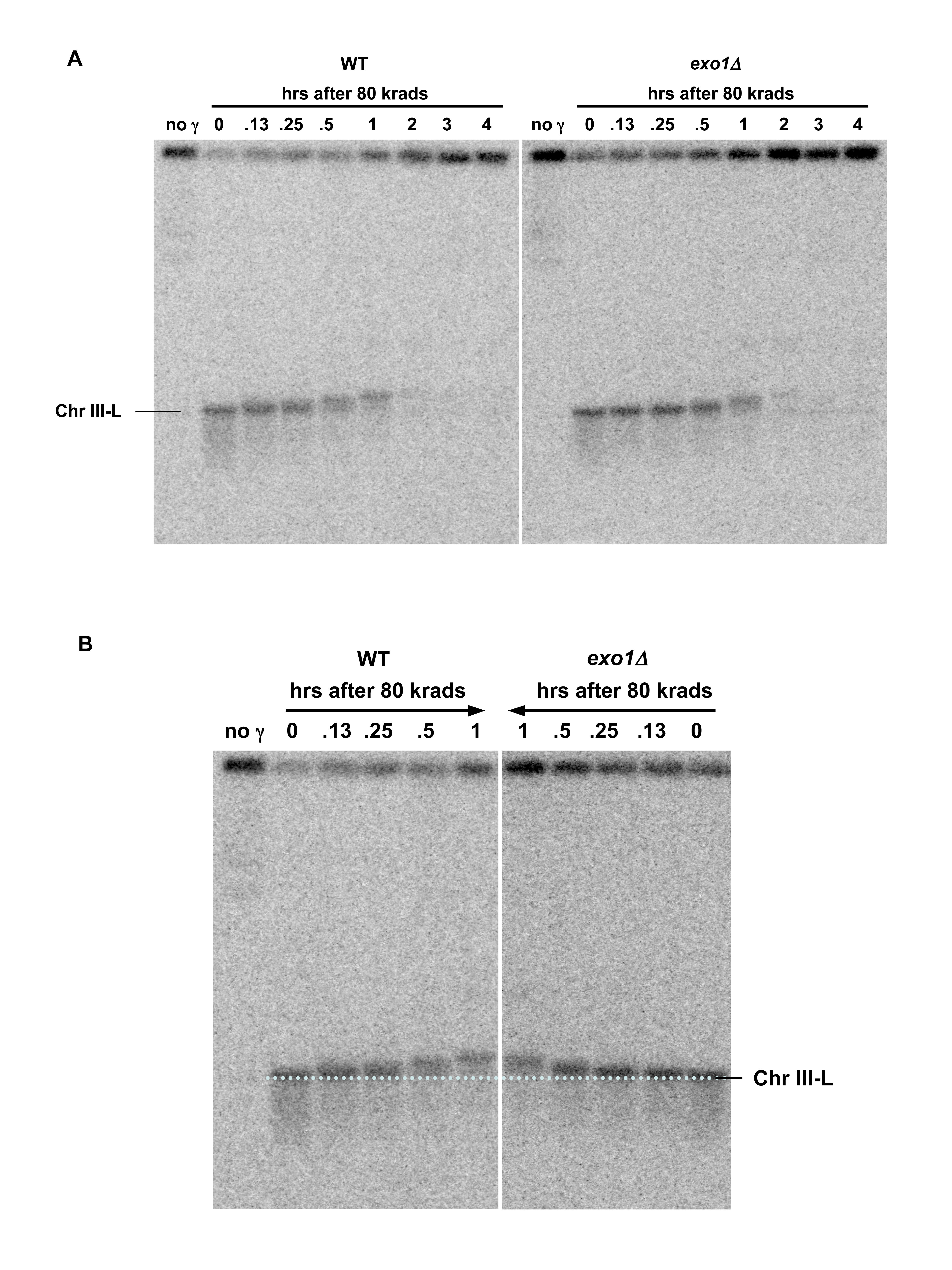

Supplement: Figure S4 — Role of EXO1 in resection at random, radiation induced DSBs. (A) Resection at a random break is slower in exo1Δ strains. Logarithmically growing WT and exo1Δ cells containing circular Chr III were arrested at G2/M, irradiated with 80 krad, and returned to YPDA as described in Figure 1A and in Materials and Methods. Samples were processed and run on PFGE (TAFE, see Materials and Methods). A Southern transfer of the TAFE gel was hybridized to the Chr III-specific CHA1 probe. While most molecules exhibited a PFGE-shift, the resection rate in exo1Δ appears to be somewhat slower than in WT, but much greater than in rad50. Chromosomal repair in the exo1 strain is comparable to that of the WT strain (Figure 1A). (B) Further comparison of resection in WT and exo1Δ strains. These images correspond to the images in Figure S4A except that the exo1Δ image (right) has been flipped horizontally to better reveal the slightly reduced PFGE shift at one hr in the exo1Δ strain vs the WT. Similar results were obtained in the exo1Δ strain containing a circular Chr V (data not shown). Although the difference in PFGE shift between the two strains at each time point is small, the actual processivity of resection could be affected as much as two-fold. Since most molecules from the exo1Δ time course exhibit PFGE shift by 1 hour, initiation of resection is not greatly affected by the absence of exonuclease 1. (2.22 MB JPG) [file pgen.1000656.s004.jpg]

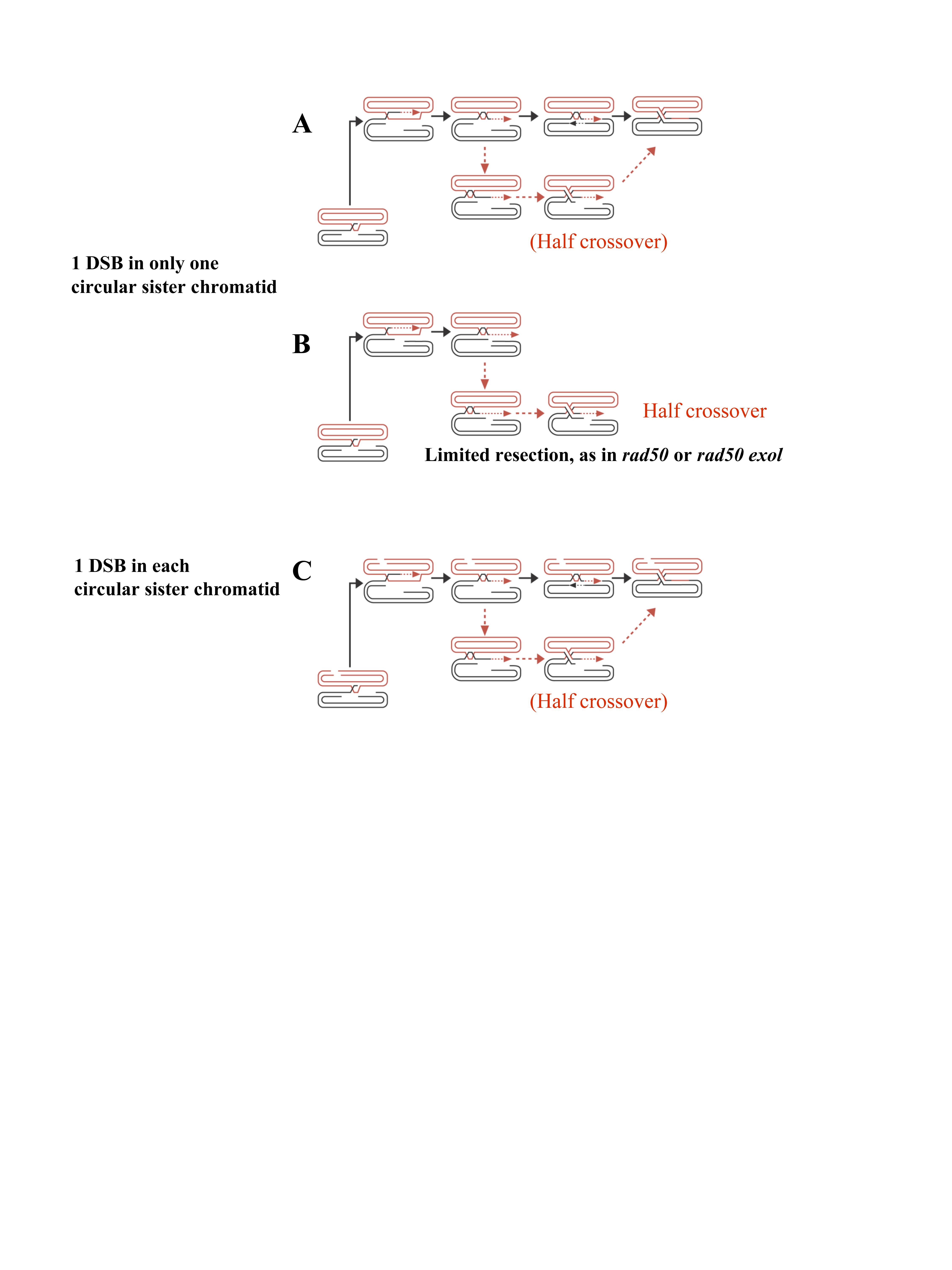

Supplement: Figure S5 — Models describing the generation of linear dimer molecules from broken circular sister chromatid. (Figures are adapted from [65] in their study of double-strand break mediated recombination in meiosis.) (A) In the wild type cells, a single DSB in one of two sister chromatids can be repaired via resection, strand invasion, strand extension by DNA synthesis, reannealing with complementary resected end and resolvase resolution of a Holiday junction to generate a circular dimer. A temporary half crossover could occur through migration of the Holiday junction and resolvase activity (lower part of (A)). (B) In cells with little or no resection of at least one end of a DSB, opportunities for completion of recombinational repair of a single DSB are reduced. Very limited resection or helicase generation of short single strand regions might allow recombinational interactions, resulting in half crossovers. (C) Pairs of sister chromatids each with a DSB could result in a linear dimer if only one is repaired. At low doses the likelihood of a cell having a single DSB in each sister chromatid is low. With increased dose, a single DSB in each chromatid becomes more likely. However, this scenario (a single DSB in each sister chromatid or two breaks in one sister and none in the other) eventually becomes less likely at increasingly higher doses that induce multiple DSBs per chromatid. (1.03 MB TIF) [file pgen.1000656.s005.tif]

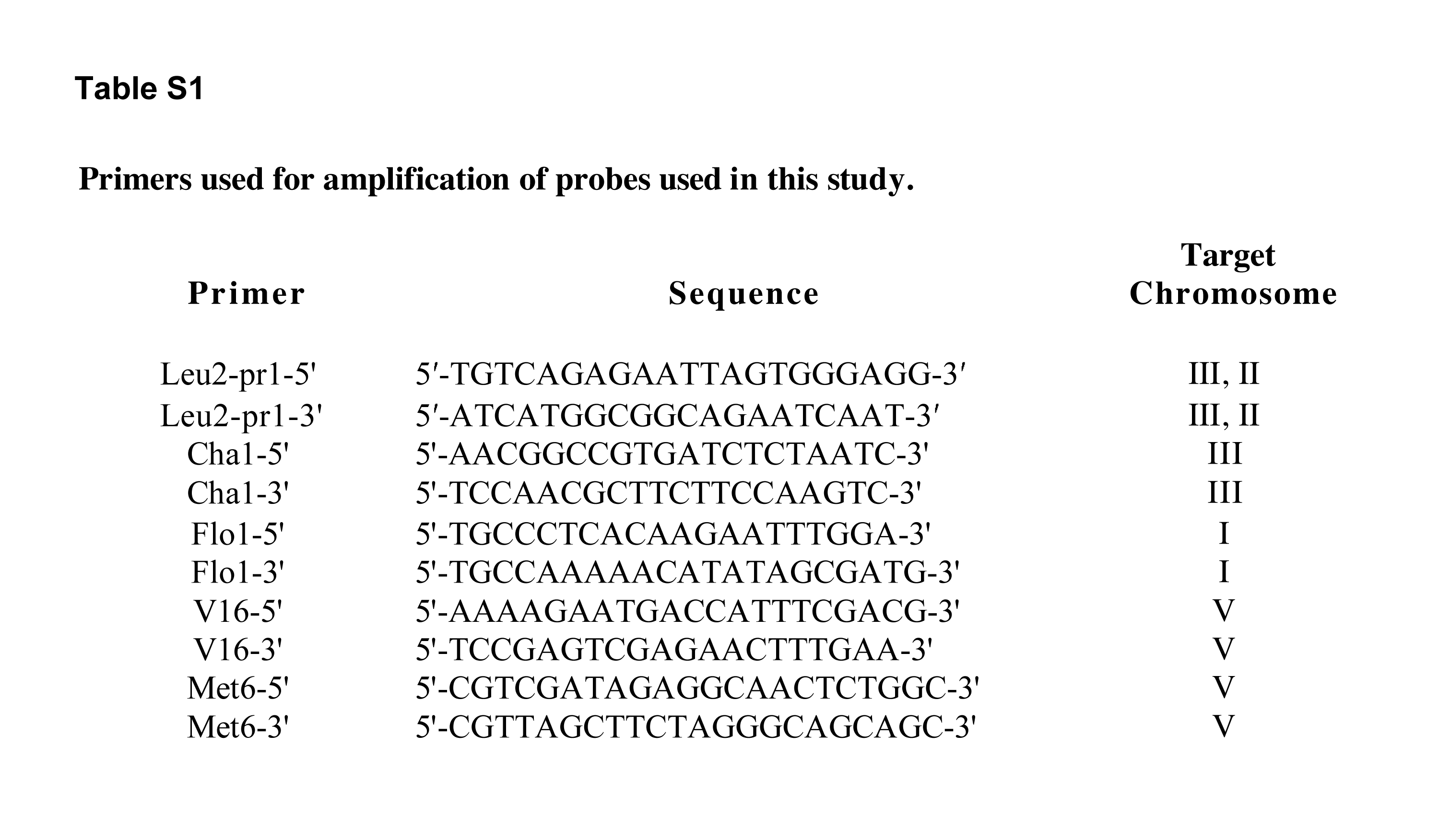

Supplement: Table S1 — Primers used for generation of probes used in this study. (0.18 MB TIF) [file pgen.1000656.s006.tif]
